# Corrector estimates in homogenization of a nonlinear transmission problem for diffusion equations in connected domains

**DOI:** 10.1002/mma.6007

**Published:** 2019-11-15

**Authors:** Victor A. Kovtunenko, Sina Reichelt, Anna V. Zubkova

**Affiliations:** ^1^ Institute for Mathematics and Scientific Computing Karl‐Franzens University of Graz, NAWI Graz Graz Austria; ^2^ Lavrentyev Institute of Hydrodynamics Siberian Division of the Russian Academy of Sciences Novosibirsk Russia; ^3^ Weierstrass Institute for Applied Analysis and Stochastics Partial Differential Equations Berlin Germany

**Keywords:** bidomain model, corrector estimates, diffusion problem, nonlinear transmission conditions, periodic unfolding technique

## Abstract

This paper is devoted to the homogenization of a nonlinear transmission problem stated in a two‐phase domain. We consider a system of linear diffusion equations defined in a periodic domain consisting of two disjoint phases that are both connected sets separated by a thin interface. Depending on the field variables, at the interface, nonlinear conditions are imposed to describe interface reactions. In the variational setting of the problem, we prove the homogenization theorem and a bidomain averaged model. The periodic unfolding technique is used to obtain the residual error estimate with a first‐order corrector.

## 1 INTRODUCTION

We consider coupled linear parabolic equations describing the diffusion of two species in two different phases of one physical domain separated by a thin periodic interface. The coupling of the species arises via nonlinear transmission conditions at the interface, which model surface reactions. Nonlinear interface reactions are relevant, for instance, in electrochemistry, see, eg, Landstorfer et al^1^ for adsorption and solvation effects at metal‐electrolyte interfaces, and Efendiev et al^2^ for electro‐chemical reactions in lithium‐ion batteries.

The characteristic length scale of the periodic cell is given by the homogenization parameter *ε*>0. The main objective is to derive a macroscopic model for vanishing *ε*, where both phases are connected sets. The limit bidomain model is given via two coupled parabolic equations defined in the macroscopic domain describing the diffusion of the two species in each phase and reactions at the interface. In the case of connected‐connected domains, we exploit the existence of a continuous extension operator from the periodic domain to the whole domain following.^3^, ^4^

A qualitative homogenization result for reaction‐diffusion systems with nonlinear transmission conditions has recently been obtained in Gahn et al.^5^ The limit in the microscopic equations is derived rigorously in the sense of the two‐scale convergence, however, without corrector estimates. There also exists a vast literature on transmission problems with linear interface conditions, eg, Donato et al^6^ and Donato and Monsurro.^7^ See references therein for the case of elliptic equations as well as the extensions of the homogenization result to parabolic equations in Jose^8^ and to nonlinear monotone transmission conditions in Donato and Le Nguyen.^9^ For the treatment of oscillating third boundary conditions, we refer to Belyaev et al^10^ and Oleinik and Shaposhnikova.^11^

Within elecktrokinetic modeling (see Allaire et al^12^), in previous studies,^13^, ^14^, ^15^, ^16^ there were considered generalized Poisson‐Nernst‐Planck (PNP) models over two‐phase domains accounting for interface reactions. The corresponding PDE system obeys a structure of the gradient flow; see, eg, other works.^17^, ^18^, ^19^ The paper^20^ considers the homogenization over a two‐phase domain for static PNP equations and homogeneous interface conditions. In Kovtunenko and Zubkova,^21^ residual error estimates for the averaged monodomain solution with first‐order correctors were justified under the simplifying assumption that the flux across the interface is of order *O*(*ε* ^2^).

In this paper, however, we are mainly interested in quantitative asymptotic results supported by corrector estimates. There exist many articles on the derivation of error estimates for different classes of reaction‐diffusion systems, eg, other works,^22^, ^23^, ^24^, ^25^ exploiting a higher regularity of the limit solution and the continuous extension operator from a perforated domain. Moreover, unfolding‐based error estimates have been proven for linear, elliptic transmission problems in Reichelt,^26^ for reaction‐diffusion systems with linear boundary conditions in perforated domains in Muntean and Reichelt,^27^ and for systems with nonlinear interface conditions in a two‐phase domain in Fatima et al.^28^ The latter results are based on the quantification of the periodicity defect for the periodic unfolding operator in Griso,^29^, ^30^ and they hold without assuming higher regularity for the corrector problem.

Our approach uses the periodic unfolding method introduced in Cioranescu et sl^31^ and further refined in Franců^32^ and Mielke and Timofte.^33^ To make our error estimates rigorous, we have to assume higher regularity for the limit solutions as well as for the correctors solving the local cell problems. This additional regularity for the limit problem is in line with established homogenization results by, eg, literature.^34^, ^35^, ^36^ Our result provides residual error estimates with a first‐order corrector of order $\sqrt{\varepsilon }$ , which is (generally) optimal for *H* ^1^‐estimates up to an Lipschitz boundary, whereas in Fatima et al,^28^ the error is of order *ε* ^1/4^. For this task, we apply the Poincaré inequality in periodic domains (see Lemma 2) and the uniform extension in connected periodic domains (see Lemma 3).

The paper is structured as follows: In Section 2, we formulate the transmission problem and all relevant assumptions. In Section 3, we prove the existence of solutions to our model and provide a priori estimates. In Sections 4 and 5, we define the periodic unfolding operator and provide important properties as well as first asymptotic results. In Section 6, we state and prove our main result on the residual error estimates.

## 2 SETTING OF THE TRANSMISSION PROBLEM

For a fixed homogenization parameter *ε*>0, we consider a macroscopic domain Ω consisting of two subsets $\Omega_{1}^{\varepsilon }$, $\Omega_{2}^{\varepsilon }$, which are disjoint by a thin interface Γ^*ε*^. The both components $\Omega_{i}^{\varepsilon }$ are assumed to be connected such that $|\partial \Omega_{i}^{\varepsilon }\cap \partial \Omega |\neq 0$. By $\left|\partial \Omega_{i}^{\varepsilon }\cap \partial \Omega \right|$, we mean the surface measure of points where the boundaries of $\Omega_{i}^{\varepsilon }$ and Ω will meet.

We make the following geometric assumptions.

(D1) The reference domain $\Omega \subset R^{d}$ is a *d*‐dimensional hyperrectangle, d⩾2, ie, it is
  $$\Omega =\prod_{k=1}^{d}(a_{k},b_{k}),\;a_{k}<b_{k}\;\text{and}\;a_{k},b_{k}\in R.$$
  This assumption suffices to split Ω into periodic cells in (D3).
(D2) The *unit cell* *Y*=(0,1)^*d*^ consists of two open, connected subsets *Y*_1_ and *Y*_2_, which have Lipschitz continuous boundaries *∂Y*_1_, *∂Y*_2_ and are disjoint by the interface Γ=*∂Y*_1_∩*∂Y*_2_. We assume the reflection symmetry, ie,
  $$\partial Y_{i}\cap \{y_{k}=0\}=\partial Y_{i}\cap \{y_{k}=1\}$$
  for *k*=1,…,*d*, *i*=1,2. This assumption allows us to define periodic functions on *Y*_*i*_ in (29). Let *n*_1_ and *n*_2_ denote the unit normal vectors at the respective boundaries *∂Y*_1_ and *∂Y*_2_. Every normal is chosen outward from the domain, and it does not depend on scaling by *ε*.
(D3) For *ε*>0, we introduce the decomposition of a point $x\in R^{d}$ as
  (1) $$x=\varepsilon \left\lfloor \frac{x}{\varepsilon }\right\rfloor +\varepsilon \left\{\frac{x}{\varepsilon }\right\}$$
  into the floor part $\left\lfloor \frac{x}{\varepsilon }\right\rfloor \in Z^{d}$ and the fractional part $\left\{\frac{x}{\varepsilon }\right\}\in Y$. According to (1), let the set of integer vectors
  $$I_{\varepsilon }=\{\lambda \in Z^{d}\;|\;\varepsilon (\lambda +y)\in \Omega \;\text{for all}\;y\in Y\}$$
  denote the numbering of local cells inside Ω. We call *ε* an admissible parameter, if the reference domain Ω from (D1) can be partitioned periodically into the local cells as follows:
  (2) $$\bar{\Omega }=\underset{\lambda \in I_{\varepsilon }}{⋃}\varepsilon (\lambda +\bar{Y}).$$
  For a treatment of small boundary layers, see Reichelt.^37^, lemma 2.3.3
(D4) As a consequence of (D1) to (D3), the periodic components $\Omega_{1}^{\varepsilon }$ and $\Omega_{2}^{\varepsilon }$ and their interface Γ^*ε*^ are determined via
  (3) $$\bar{\Omega_{i}^{\varepsilon }}=\underset{\lambda \in I_{\varepsilon }}{⋃}\bar{Y_{i}^{\lambda }},\;Y_{i}^{\lambda }=\varepsilon (\lambda +Y_{i}),\;\Gamma^{\varepsilon }=\partial \Omega_{1}^{\varepsilon }\cap \partial \Omega_{2}^{\varepsilon }.$$
  By this, the outward normal vectors $n_{i}^{\varepsilon }$ at $\partial \Omega_{i}^{\varepsilon }$ coincide with the normal vectors *n*_*i*_ at *∂Y*_*i*_ for *i*=1,2 and do not depend on the scaling *ε*. The interface Γ^*ε*^ is a Lipschitz continuous manifold.

For admissible *ε*>0, time *t*∈(0,*T*) with the final time *T*>0 fixed, the space variable $x\in \bar{\Omega_{1}^{\varepsilon }}⋃\bar{\Omega_{2}^{\varepsilon }}$ in the two‐component domain, we consider a nonlinear transmission problem for $u_{i}^{\varepsilon }(t,x)$, *i*=1,2, such that

(4a) $$\partial_{t}u_{i}^{\varepsilon }-\text{div}(A_{i}^{\varepsilon }\nabla u_{i}^{\varepsilon })=0\;\text{in}\;\Omega_{i}^{\varepsilon },$$

(4b) $$A_{i}^{\varepsilon }\nabla u_{i}^{\varepsilon }\cdot n_{i}=\varepsilon g_{i}(u_{1}^{\varepsilon },u_{2}^{\varepsilon })\;\text{on}\;\Gamma^{\varepsilon },$$

(4c) $$\;u_{i}^{\varepsilon }=0\;\text{on}\;\partial \Omega_{i}^{\varepsilon }\cap \partial \Omega ,$$

(4d) $$\;u_{i}^{\varepsilon }=u_{i}^{\text{in}}\;\text{as}\;t=0.$$

The notation *∂*_*t*_ stands for the time derivative, ∇ for the spatial gradient, and *“*  · ^*′′*^ for the scalar product in $R^{d}$. Below, we explain in detail the terms entering the system (4). We note that |Γ^*ε*^|=*O*(1/*ε*); therefore, the scaling *ε* in (4b) appears naturally just compensating the longer interface.

(A1) The diffusivity matrices $A_{i}(y)\in L^{\infty }(Y_{i};R_{sym}^{d\times d})$, *i*=1,2, are symmetric, uniformly bounded and elliptic: There exist 0<α⩽β such that
  (5) $$\alpha |\xi {|}^{2}⩽A_{i}(y)\xi \cdot \xi ⩽\beta |\xi {|}^{2}\;\text{for all}\;\xi \in R^{d},\;\text{a.e.}\;y\in Y_{i}.$$

The matrices entering (4a) to (4c) are defined as $A_{i}^{\varepsilon }(x)=A_{i}\left(\left\{\frac{x}{\varepsilon }\right\}\right)$ according to the notation (1) and are assumed to be periodic.

In the transmission conditions (4b), the functions $g_{i}:R^{2}\mapsto R$, *i*=1,2, describe interface reactions and are assumed to satisfy

(G1) the uniform growth condition: there exists *K*_g_>0 such that
  (6) $$|g_{i}(u_{1},u_{2})|⩽K_{g},\;\text{for all}\;u_{1},u_{2}\in R;$$
(G2) the Lipschitz continuity: There exists $L_{g}⩾0$ such that
  (7) $$|g_{i}(u_{1},u_{2})-g_{i}(v_{1},v_{2})|⩽L_{g}\left(\left|u_{1}-v_{1}|+|u_{2}-v_{2}\right|\right),$$
  for all $u_{i},v_{i}\in R$, *i*=1,2.

The linear diffusion equations (4a) are supported by the standard, homogeneous Dirichlet boundary conditions (4c) and the initial data (4d) for given $u_{i}^{\text{in}}\in L^{2}(\Omega )$, *i*=1,2.

We introduce the variational formulation of the problem (4) as follows: find $u_{i}^{\varepsilon }\in U_{i}^{\varepsilon }$, *i*=1,2, in the search (solution) space

$$U_{i}^{\varepsilon }=\{u\in C(0,T;L^{2}(\Omega_{i}^{\varepsilon }))\cap L^{2}(0,T;H^{1}(\Omega_{i}^{\varepsilon })):\;\partial_{t}u\in L^{2}(0,T;H^{1}{\left(\Omega_{i}^{\varepsilon }\right)}^{*}),\;u=0\;\text{on}\;\partial \Omega_{i}^{\varepsilon }\cap \partial \Omega \},$$

satisfying the initial condition (4d) and the nonlinear equation

(8) $$\int_{0}^{T}\left({\left\langle \partial_{t}u_{i}^{\varepsilon },v_{i}\right\rangle }_{\Omega_{i}^{\varepsilon }}+\int_{\Omega_{i}^{\varepsilon }}A_{i}^{\varepsilon }\nabla u_{i}^{\varepsilon }\cdot \nabla v_{i}\;dx\right)\;dt=\int_{0}^{T}\int_{\Gamma^{\varepsilon }}\varepsilon g_{i}(u_{1}^{\varepsilon },u_{2}^{\varepsilon })v_{i}\;d\sigma_{x}\;dt,$$

for all test functions *v*_*i*_ from the test space

$$V_{i}^{\varepsilon }:=\{v\in L^{2}(0,T;H^{1}(\Omega_{i}^{\varepsilon })),\;v=0\;\text{on}\;\partial \Omega_{i}^{\varepsilon }\cap \partial \Omega \}.$$

The notation $H^{1}{\left(\Omega_{i}^{\varepsilon }\right)}^{*}$ in $U_{i}^{\varepsilon }$ stands for the topologically dual space to $H^{1}(\Omega_{i}^{\varepsilon })$, and ${\left\langle \cdot ,\cdot \right\rangle }_{\Omega_{i}^{\varepsilon }}$ denotes the duality between them.

## 3 WELL‐POSEDNESS

This section provides the existence of weak solutions in the sense of variational formulation for the microscopic problem (8).

Theorem 1 (Well‐posedness)

(i) The unique solution $u_{i}^{\varepsilon }\in U_{i}^{\varepsilon }$ to the nonlinear transmission problem (8) exists and satisfies the following a priori estimate:
  (9) $$\begin{array}{ll} \|u_{i}^{\varepsilon }{\|}_{U_{i}^{\varepsilon }}^{2} & :=\|u_{i}^{\varepsilon }{\|}_{C(0,T;L^{2}(\Omega_{i}^{\varepsilon }))}^{2}+\|u_{i}^{\varepsilon }{\|}_{L^{2}(0,T;H^{1}(\Omega_{i}^{\varepsilon }))}^{2}+\|\partial_{t}u_{i}^{\varepsilon }{\|}_{L^{2}(0,T;H^{1}{\left(\Omega_{i}^{\varepsilon }\right)}^{*})}^{2}\\ & ⩽C_{1}\|u_{i}^{\text{in}}{\|}_{L^{2}(\Omega_{i}^{\varepsilon })}^{2}+C_{2}K_{g}^{2}+C_{3},\;C_{1},C_{2},C_{3}⩾0, \end{array}$$
  uniformly in *ε*∈(0,*ε*_0_) for *ε*_0_>0 sufficiently small.
(ii) Under assumptions on positivity of the initial data $u_{i}^{\text{in}}>0$ everywhere in $\bar{\Omega }$, the solution $u_{i}^{\varepsilon }$ is positive at least locally in time, and $u_{i}^{\varepsilon }⩾0$ at any time under the assumption of the positive production rate from RoubÍček^38^:
  (10) $$g_{i}(u_{1}^{\varepsilon },u_{2}^{\varepsilon }){\left(u_{i}^{\varepsilon }\right)}^{-}=0,$$
  where ${\left(u_{i}^{\varepsilon }\right)}^{-}=-\min(0,u_{i}^{\varepsilon })$ stands for the negative part of the function.

(i) To prove existence of the solution, we apply the Tikhonov‐Schauder fixed point theorem. We iterate (8) starting with the suitable initialization $u_{i}^{m_{0}}=u_{i}^{\text{in}}$, $m_{0}\in N$, *i*=1,2.
  For *m*>*m*_0_, m∈N, a solution $u_{i}^{m}\in U_{i}^{\varepsilon }$ can be found, which satisfies the initial data (4d) and the linearized equations
  (11) $$\int_{0}^{T}\left({\left\langle \partial_{t}u_{i}^{m},v_{i}\right\rangle }_{\Omega_{i}^{\varepsilon }}+\int_{\Omega_{i}^{\varepsilon }}A_{i}^{\varepsilon }\nabla u_{i}^{m}\cdot \nabla v_{i}\;dx\right)\;dt=\int_{0}^{T}\int_{\Gamma^{\varepsilon }}\varepsilon g_{i}^{m-1}v_{i}\;d\sigma_{x}\;dt,$$
  for all test functions $v_{i}\in V_{i}^{\varepsilon }$, using the notation $g_{i}^{m-1}:=g_{i}(u_{1}^{m-1},u_{2}^{m-1})$ for short. We can test (11) with $v_{i}=u_{i}^{m}$ leading to
  (12) $$\int_{0}^{T}\left({\left\langle \partial_{t}u_{i}^{m},u_{i}^{m}\right\rangle }_{\Omega_{i}^{\varepsilon }}+\int_{\Omega_{i}^{\varepsilon }}A_{i}^{\varepsilon }\nabla u_{i}^{m}\cdot \nabla u_{i}^{m}\;dx\right)\;dt=\int_{0}^{T}\int_{\Gamma^{\varepsilon }}\varepsilon g_{i}^{m-1}u_{i}^{m}\;d\sigma_{x}\;dt.$$
  We estimate the integral in the right‐hand side of (12) applying weighted Young inequality with a weight $\frac{2\delta }{K_{\text{tr}}}>0$, the trace theorem (25) below, and the growth condition (6):
  (13) $$\left|\int_{0}^{T}\int_{\Gamma^{\varepsilon }}\varepsilon g_{i}^{m-1}u_{i}^{m}\;d\sigma_{x}\;dt\right|⩽\frac{\delta \varepsilon }{K_{\text{tr}}}\int_{0}^{T}\int_{\Gamma^{\varepsilon }}{\left(u_{i}^{m}\right)}^{2}\;d\sigma_{x}\;dt+\frac{\varepsilon K_{\text{tr}}}{4\delta }\int_{0}^{T}\int_{\Gamma^{\varepsilon }}{\left(g_{i}^{m-1}\right)}^{2}\;d\sigma_{x}\;dt⩽\delta \|u_{i}^{m}{\|}_{L^{2}(0,T;H^{1}(\Omega_{i}^{\varepsilon }))}^{2}+C,$$
  where $C=\frac{K_{\text{tr}}}{4\delta }K_{g}^{2}T\varepsilon |\Gamma^{\varepsilon }|=O(1)$ with a constant *K*_tr_ from the trace theorem (25) and *K*_g_ from (6). Expressing the first term in the left‐hand side of (12) by the chain rule as $\langle \partial_{t}u_{i}^{m},u_{i}^{m}\rangle =\frac{1}{2}\frac{d}{dt}\|u_{i}^{m}{\|}_{L^{2}(\Omega_{i}^{\varepsilon })}$, using the uniform ellipticity (5) of $A_{i}^{\varepsilon }$ and the estimate (13), this follows
  (14) $$\frac{1}{2}\frac{d}{dt}\int_{0}^{T}\int_{\Omega_{i}^{\varepsilon }}{\left(u_{i}^{m}\right)}^{2}\;dx\;dt+(\alpha -\delta )\|\nabla u_{i}^{m}{\|}_{L^{2}{\left(0,T;L^{2}(\Omega_{i}^{\varepsilon })\right)}^{d}}^{2}⩽\delta \|u_{i}^{m}{\|}_{L^{2}(0,T;L^{2}(\Omega_{i}^{\varepsilon }))}^{2}+C.$$
  For *δ*<*α*, applying Grönwall inequality, we obtain
  (15) $$\|u_{i}^{m}(t){\|}_{L^{2}(\Omega_{i}^{\varepsilon })}^{2}+\frac{C}{\delta }⩽\left(\|u_{i}^{in}{\|}_{L^{2}(\Omega_{i}^{\varepsilon })}^{2}+\frac{C}{\delta }\right)e^{2\delta t}\;\text{for}\;t\in (0,T),$$
  and taking in (14) the supremum over *t*∈(0,*T*), we conclude
  $$\|u_{i}^{m}{\|}_{L^{\infty }(0,T;L^{2}(\Omega_{i}^{\varepsilon }))}^{2}+\|u_{i}^{m}{\|}_{L^{2}(0,T;H^{1}(\Omega_{i}^{\varepsilon }))}^{2}⩽C_{1}\|u_{i}^{\text{in}}{\|}_{L^{2}(\Omega_{i}^{\varepsilon })}^{2}+C_{2}K_{g}^{2}+C_{3},\;C_{1},C_{2},C_{3}⩾0.$$
  Hence, using (6) from (12), it follows $\left\|\partial_{t}u_{i}^{m}{\|}_{L^{2}(0,T;H^{1}{\left(\Omega_{i}^{\varepsilon }\right)}^{*})}^{2}=O(1\right)$ uniformly with respect to *m*→*∞* and *ε*→0, and the continuous embedding of the solution in $C(0,T;L^{2}(\Omega_{i}^{\varepsilon }))$ holds; see Dautray and Lions.^39^, p509
  Therefore, the mapping $M:U_{i}^{\varepsilon }\mapsto U_{i}^{\varepsilon }$ defined when solving (11) has compact image, and hence, there exists an accumulation point $u_{i}^{\varepsilon }\in U_{i}^{\varepsilon }$, *i*=1,2, and a subsequence still denoted by *m* such that as *m*→*∞*
  $$u_{i}^{m}⇀u_{i}^{\varepsilon }\;\text{weakly in}\;U_{i}^{\varepsilon }\;\text{and}\;u_{i}^{m}\to u_{i}^{\varepsilon }\;\text{strongly in}\;L^{2}(0,T;L^{2}(\Gamma^{\varepsilon })).$$
  The continuity of M in the weak topology is justified using the Lipschitz continuity of the nonlinear term *g*_*i*_ in (7). Applying the fixed point theorem^40^, section 4.8, theorem 8.1, p293 and the a priori estimate (9) proves the existence of a weak solution of problem (8).
  To prove uniqueness, we consider the difference $w_{i}^{\varepsilon }:=u_{i}^{1,\varepsilon }-u_{i}^{2,\varepsilon }$, *i*=1,2, of two solutions of (8) with the test function $v_{i}=w_{i}^{\varepsilon }$:
  (16) $$\frac{1}{2}\int_{\Omega_{i}^{\varepsilon }}{\left.{\left(w_{i}^{\varepsilon }\right)}^{2}\right|}_{t=0}^{T}\;dx+\int_{0}^{T}\int_{\Omega_{i}^{\varepsilon }}A_{i}^{\varepsilon }\nabla w_{i}^{\varepsilon }\cdot \nabla w_{i}^{\varepsilon }\;dx\;dt=I_{g_{i}}^{\varepsilon },\;I_{g_{i}}^{\varepsilon }:=\int_{0}^{T}\int_{\Gamma^{\varepsilon }}\varepsilon \left(g_{i}(u_{1}^{1,\varepsilon },u_{2}^{1,\varepsilon })-g_{i}(u_{1}^{2,\varepsilon },u_{2}^{2,\varepsilon })\right)w_{i}^{\varepsilon }\;d\sigma_{x}\;dt.$$
  The integral $I_{g_{i}}^{\varepsilon }$ is estimated due to the Lipschitz continuity (7) as
  (17) $$|I_{g_{i}}^{\varepsilon }|⩽\varepsilon L_{g}\int_{0}^{T}\int_{\Gamma^{\varepsilon }}(|w_{1}^{\varepsilon }{|}^{2}+|w_{2}^{\varepsilon }{|}^{2})w_{i}^{\varepsilon }\;d\sigma_{x}\;dt.$$
  Then, collecting the expressions (16) and (17), applying the Cauchy‐Schwarz and Grönwall inequalities, we get
  $$\sum_{i=1}^{2}\|w_{i}^{\varepsilon }(t){\|}^{2}⩽\sum_{i=1}^{2}\|w_{i}^{\varepsilon }(0){\|}^{2}e^{4K_{\text{tr}}L_{g}t}=0$$
  and hence conclude $w_{i}^{\varepsilon }\equiv 0$, which proves $u_{i}^{1,\varepsilon }\equiv u_{i}^{2,\varepsilon }$.
(ii) To prove the nonnegativity of $u_{i}^{\varepsilon }$, we decompose the solution into the positive and the negative parts as: $u_{i}^{\varepsilon }={\left(u_{i}^{\varepsilon }\right)}^{+}-{\left(u_{i}^{\varepsilon }\right)}^{-}$ and substitute it in the Equation (8) with the test function $v_{i}={\left(u_{i}^{\varepsilon }\right)}^{-}$. The assumption of the positive production rate (10) together with the uniform ellipticity (5) of $A_{i}^{\varepsilon }$ and the nonnegativity of the initial data lead to the estimate:
  $$\underset{t\in (0,T)}{\sup}\frac{1}{2}\int_{\Omega_{i}^{\varepsilon }}{\left({\left(u_{i}^{\varepsilon }\right)}^{-}\right)}^{2}\;dx+\alpha \|\nabla {\left(u_{i}^{\varepsilon }\right)}^{-}{\|}_{L^{2}{\left(0,T;L^{2}(\Omega_{i}^{\varepsilon })\right)}^{d}}^{2}⩽\frac{1}{2}\int_{\Omega_{i}^{\varepsilon }}{\left.{\left({\left(u_{i}^{\varepsilon }\right)}^{-}\right)}^{2}\right|}_{t=0}\;dx=0;$$
  hence, ${\left(u_{i}^{\varepsilon }\right)}^{-}\equiv 0$ and $u_{i}^{\varepsilon }⩾0$. If $u_{i}^{\varepsilon }(0)=u_{i}^{in}>0$ everywhere in $\bar{\Omega }$, then $u_{i}^{\varepsilon }(t)>0$ at least for *t* sufficiently small, which follows by the continuity of the solution. This completes the proof.

□

We note that Theorem 1 can be extended for inhomogeneous diffusion equations (4a), where the uniform upper bound is proved in Gurevich and Reichelt^41^ for reaction functions distributed over domains $\Omega_{i}^{\varepsilon }$.

## 4 PERIODIC UNFOLDING TECHNIQUE

Following Cioranescu et al,^42^ we recall the technique based on the periodic unfolding and averaging operators providing continuous mappings between the components $\bar{\Omega_{i}^{\varepsilon }}$ and $\bar{Y_{i}}$, i = 1,2, up to the boundaries.

Definition 1 For $u(x)\in L^{2}(\Omega_{i}^{\varepsilon })$, the unfolding operator $T_{\varepsilon }:L^{2}(\Omega_{i}^{\varepsilon })\mapsto L^{2}(\Omega ;L^{2}(Y_{i}))$, *i*=1,2, in the domain is defined by

(18a) $$(T_{\varepsilon }u)(x,y):=u\left(\varepsilon \left\lfloor \frac{x}{\varepsilon }\right\rfloor +\varepsilon y\right),\;\text{for}\;x\in \Omega \;\text{and}\;y\in Y_{i},$$

and for $u(x)\in L^{2}(\partial \Omega_{i}^{\varepsilon })$, the operator $T_{\varepsilon }:L^{2}(\partial \Omega_{i}^{\varepsilon })\mapsto L^{2}(\Omega ;L^{2}(\partial Y_{i}))$, *i*=1,2, is performed on the boundary by

(18b) $$(T_{\varepsilon }u)(x,y):=u\left(\varepsilon \left\lfloor \frac{x}{\varepsilon }\right\rfloor +\varepsilon y\right),\;\text{for}\;x\in \Omega \;\text{and}\;y\in \partial Y_{i}.$$

For *φ*(*x*,*y*)∈*L* ^2^(Ω;*L* ^2^(*Y*_*i*_)), the averaging operator $T_{\varepsilon }^{-1}:L^{2}(\Omega ;L^{2}(Y_{i}))\mapsto L^{2}(\Omega_{i}^{\varepsilon })$, *i*=1,2, in the domain is defined by

(19a) $$(T_{\varepsilon }^{-1}\varphi )(x):=\frac{1}{\left|Y\right|}\int_{Y_{i}}\varphi \left(\varepsilon \left\lfloor \frac{x}{\varepsilon }\right\rfloor +\varepsilon z,\left\{\frac{x}{\varepsilon }\right\}\right)\;dz,\;\text{for}\;x\in \Omega_{i}^{\varepsilon },$$

and for *φ*(*x*,*y*)∈*L* ^2^(Ω;*L* ^2^(*∂Y*_*i*_)), the operator $T_{\varepsilon }^{-1}:L^{2}(\Omega ;L^{2}(\partial Y_{i}))\mapsto L^{2}(\partial \Omega_{i}^{\varepsilon })$, *i*=1,2, on the boundary is expressed by

(19b) $$(T_{\varepsilon }^{-1}\varphi )(x):=\frac{1}{\left|Y\right|}\int_{Y_{i}}\varphi \left(\varepsilon \left\lfloor \frac{x}{\varepsilon }\right\rfloor +\varepsilon z,\left\{\frac{x}{\varepsilon }\right\}\right)\;dz,\;\text{for}\;x\in \partial \Omega_{i}^{\varepsilon }.$$

Abusing the notation $T_{\varepsilon }^{-1}$ is used for a left inverse operator of *T*_*ε*_ according to Lemma 1 (i), which is also right inverse in the special cases accounting in Lemma 1 (ii). For those functions that belong to $H^{1}(\Omega_{i}^{\varepsilon })$, the restriction of the unfolding operator *T*_*ε*_ is well‐defined as the mapping $H^{1}(\Omega_{i}^{\varepsilon })\mapsto L^{2}(\Omega ;H^{1}(Y_{i}))$, and for functions in *L* ^2^(Ω;*H* ^1^(*Y*_*i*_)), the restriction of the averaging operator $T_{\varepsilon }^{-1}$ is well‐defined as $L^{2}(\Omega ;H^{1}(Y_{i}))\mapsto H^{1}(\underset{\lambda \in I_{\varepsilon }}{⋃}Y_{i}^{\lambda })$, where $Y_{i}^{\lambda }$ is from (3). We note that the spaces $H^{1}(\underset{\lambda \in I_{\varepsilon }}{⋃}Y_{i}^{\lambda })$ and $H^{1}(\Omega_{i}^{\varepsilon })$ do not coincide because functions from $H^{1}(\underset{\lambda \in I_{\varepsilon }}{⋃}Y_{i}^{\lambda })$ are discontinuous while they can have jumps across the interface Γ^*ε*^.

The operator properties are collected below in Lemma 1.

Lemma 1 (Properties of the operators *T*_*ε*_ and $T_{\varepsilon }^{-1}$) For arbitrary $x\mapsto u(x)\in H^{1}(\Omega_{i}^{\varepsilon })\cap L^{2}(\partial \Omega_{i}^{\varepsilon })$ and (*x*,*y*)↦*φ*(*x*,*y*)∈*L* ^2^(Ω;*H* ^1^(*Y*_*i*_)∩*L* ^2^(*∂Y*_*i*_)), *i*=1,2, and the extension by zero: ū(x)=u(x) for $x\in \Omega_{i}^{\varepsilon }$, otherwise ū(x)=0 for $x\in \Omega \setminus \bar{\Omega_{i}^{\varepsilon }}$, the following properties hold:

(i) invertibility of *T*_*ε*_: $\left(T_{\varepsilon }^{-1}T_{\varepsilon })u(x)=u(x\right)$;
(ii) invertibility of $T_{\varepsilon }^{-1}$:
  (iia) $\left(T_{\varepsilon }T_{\varepsilon }^{-1}\varphi )(x,y)=\varphi (y\right)$ for *x*∈Ω, if *φ*(*y*) is a constant or periodic function of the argument *y*∈*Y*_*i*_,
  (iib) $\left(T_{\varepsilon }T_{\varepsilon }^{-1}ū)(x,\;\cdot \;)=(T_{\varepsilon }^{-1}ū)(x)=\frac{\left|Y_{i}\right|}{\left|Y\right|}{\left\langle T_{\varepsilon }u\right\rangle }_{Y_{i}}(x\right)$ for *x*∈Ω, where is the average ${\left\langle \;\cdot \;\right\rangle }_{Y_{i}}=\frac{1}{\left|Y_{i}\right|}\int_{Y_{i}}\;(\cdot )\;dy$;
(iii) composition rule: $T_{\varepsilon }(F(u))(x,y)=F(T_{\varepsilon }u)(x,y)$ for any elementary function F;
(iv) chain rules: *εT*_*ε*_(∇*u*)(*x*,*y*)=∇_*y*_(*T*_*ε*_ *u*)(*x*,*y*), and $\nabla (T_{\varepsilon }^{-1}\varphi )(x)=T_{\varepsilon }^{-1}(\nabla \varphi +\frac{1}{\varepsilon }\nabla_{y}\varphi )(x)$ for $x\in Y_{i}^{\lambda }$ and *φ*∈*H* ^1^(Ω×*Y*_*i*_);
(v) integration rules:
  (20a) $$\int_{\Omega_{i}^{\varepsilon }}u(x)\;dx=\frac{1}{\left|Y\right|}\int_{\Omega \times Y_{i}}(T_{\varepsilon }u)(x,y)\;dx\;dy,$$
  (20b) $$\int_{\partial \Omega_{i}^{\varepsilon }}u(x)\;d\sigma_{x}=\frac{1}{\varepsilon |Y|}\int_{\Omega \times \partial Y_{i}}(T_{\varepsilon }u)(x,y)\;dx\;d\sigma_{y};$$
(vi) boundedness of *T*_*ε*_:
  (21a) $$\int_{\Omega_{i}^{\varepsilon }}u^{2}(x)\;dx=\frac{1}{\left|Y\right|}\int_{\Omega \times Y_{i}}{\left(T_{\varepsilon }u\right)}^{2}(x,y)\;dx\;dy,$$
  (21b) $$\int_{\Omega_{i}^{\varepsilon }}|\nabla u{|}^{2}(x)\;dx=\frac{1}{\varepsilon^{2}|Y|}\int_{\Omega \times Y_{i}}|\nabla_{y}(T_{\varepsilon }u){|}^{2}(x,y)\;dx\;dy,$$
  (21c) $$\int_{\partial \Omega_{i}^{\varepsilon }}u^{2}(x)\;d\sigma_{x}=\frac{1}{\varepsilon |Y|}\int_{\Omega \times \partial Y_{i}}{\left(T_{\varepsilon }u\right)}^{2}(x,y)\;dx\;d\sigma_{y}.$$

The property (iib) follows in a straightforward manner from the calculation of $\left(T_{\varepsilon }T_{\varepsilon }^{-1}ū)(x,z)=(T_{\varepsilon }^{-1}ū)(x\right)$ for *x*∈Ω and *z*∈*Y*:

$$\frac{1}{\left|Y\right|}\int_{Y_{i}}ū\left(\varepsilon \left\lfloor \frac{\varepsilon \left\lfloor \frac{x}{\varepsilon }\right\rfloor +\varepsilon z}{\varepsilon }\right\rfloor +\varepsilon y\right)\;dy=\frac{1}{\left|Y\right|}\int_{Y_{i}}ū\left(\varepsilon \left\lfloor \frac{x}{\varepsilon }\right\rfloor +\varepsilon y\right)\;dy=T_{\varepsilon }^{-1}ū(x)$$

and the fact that $T_{\varepsilon }^{-1}ū=\frac{\left|Y_{i}\right|}{\left|Y\right|}{\left\langle T_{\varepsilon }u\right\rangle }_{Y_{i}}$ as a consequence of the definition (19a) if φ(x,y)≡ū(x) for all *φ*(*x*,*y*)∈*L* ^2^(Ω;*H* ^1^(*Y*_*i*_)). The proof of the other properties can be found in other studies.^20^, ^21^, ^31^, ^42^, ^43^

□

## 5 ASYMPTOTIC ANALYSIS

In this section, we collect some auxiliary tools used later in the derivation of the residual error estimates.

Lemma 2 (Poincaré inequality in periodic domains) For $u(x)\in H^{1}(\Omega_{i}^{\varepsilon })$, the following Poincaré inequality holds (see, eg, Cioranescu et al^42^, ^43^):

(22) $$\|u-{\left\langle T_{\varepsilon }u\right\rangle }_{Y_{i}}{\|}_{L^{2}(\Omega_{i}^{\varepsilon })}^{2}⩽\varepsilon^{2}K_{\;P}\|\nabla u{\|}_{L^{2}{\left(\Omega_{i}^{\varepsilon }\right)}^{d}}^{2},\;K_{\;P}>0.$$

We recall the Poincaré inequality for a function *φ*(*y*)∈*H* ^1^(*Y*_*i*_) in the unit cell with connected subsets *Y*_*i*_ for *i*=1,2:

(23) $$\int_{Y_{i}}{\left(\varphi -{\left\langle \varphi \right\rangle }_{Y_{i}}\right)}^{2}\;dy⩽K_{\;P}\int_{Y_{i}}|\nabla_{y}\varphi {|}^{2}\;dy,\;{\left\langle \varphi \right\rangle }_{Y_{i}}:=\frac{1}{\left|Y_{i}\right|}\int_{Y_{i}}\varphi (y)\;dy.$$

Integrating (23) over Ω yields

$$\int_{\Omega \times Y_{i}}|\varphi -{\left\langle \varphi \right\rangle }_{Y_{i}}{|}^{2}\;dx\;dy⩽K_{\;P}\int_{\Omega \times Y_{i}}|\nabla_{y}\varphi {|}^{2}\;dx\;dy$$

for all *φ*∈*L* ^2^(Ω;*H* ^1^(*Y*_*i*_)). Choosing *φ*=*T*_*ε*_ *u* gives

$$\begin{array}{ccc} \frac{1}{\left|Y\right|}\int_{\Omega \times Y_{i}}|T_{\varepsilon }u-{\left\langle T_{\varepsilon }u\right\rangle }_{Y_{i}}{|}^{2}\;dx\;dy & ⩽\frac{K_{P}}{\left|Y\right|}\int_{\Omega \times Y_{i}}|\nabla_{y}(T_{\varepsilon }u){|}^{2}\;dx\;dy & \\ & =K_{P}\varepsilon^{2}\|\nabla u{\|}_{L^{2}(\Omega_{i}^{\varepsilon })}^{2}. \end{array}$$

For the left‐hand side, we use the composition rule (iii) as well as $T_{\varepsilon }{\left\langle T_{\varepsilon }u\right\rangle }_{Y_{i}}={\left\langle T_{\varepsilon }u\right\rangle }_{Y_{i}}$. For all (*x*,*y*)∈Ω×*Y*_*i*_, we have

$$\begin{array}{ccc} & \;\left(T_{\varepsilon }{\left\langle T_{\varepsilon }u\right\rangle }_{Y_{i}}\right)(x,y)=\left(T_{\varepsilon }\left((x,z)\mapsto \frac{1}{\left|Y\right|}\int_{Y_{i}}u\left(\varepsilon \left\lfloor \frac{x}{\varepsilon }\right\rfloor +\varepsilon z\right)\;dz\right)\right)(x,y) & \\ & =\frac{1}{\left|Y\right|}\int_{Y_{i}}u\left(\varepsilon \left\lfloor \frac{\varepsilon \left\lfloor \frac{x}{\varepsilon }\right\rfloor +\varepsilon y}{\varepsilon }\right\rfloor +\varepsilon z\right)\;dz=\frac{1}{\left|Y\right|}\int_{Y_{i}}u\left(\varepsilon \left\lfloor \frac{x}{\varepsilon }\right\rfloor +\varepsilon z\right)\;dz={\left\langle T_{\varepsilon }u\right\rangle }_{Y_{i}}(x), \end{array}$$

while noting that $\left\lfloor \frac{\varepsilon \left\lfloor \frac{x}{\varepsilon }\right\rfloor +\varepsilon y}{\varepsilon }\right\rfloor =\left\lfloor \frac{x}{\varepsilon }\right\rfloor$ for all *y*∈(0,1)^*d*^. This shows, in particular, that $y\mapsto \left(T_{\varepsilon }{\left\langle T_{\varepsilon }u\right\rangle }_{Y_{i}}\right)(x,y)$ is constant for a.e. *x*∈Ω.

□

We recall the trace theorem in unit cells for a function *φ*∈*L* ^2^(Ω;*H* ^1^(*Y*_*i*_)):

(24) $$\|\varphi {\|}_{L^{2}(\partial Y_{i})}^{2}⩽K_{\text{tr}}(\|\varphi {\|}_{L^{2}(Y_{i})}^{2}+\|\nabla_{y}\varphi {\|}_{L^{2}{\left(Y_{i}\right)}^{d}}^{2})=K_{\text{tr}}\|\varphi {\|}_{H^{1}(Y_{i})}^{2},$$

with *K*_tr_>0. After the substitution of *φ*=*T*_*ε*_ *u* for the function $u(x)\in H^{1}(\Omega_{i}^{\varepsilon })$, there follows (see, eg, Monsurrò^44^):

(25) $$\|u{\|}_{L^{2}(\partial \Omega_{i}^{\varepsilon })}^{2}⩽K_{\text{tr}}\left(\frac{1}{\varepsilon }\|u{\|}_{L^{2}(\Omega_{i}^{\varepsilon })}^{2}+\varepsilon \|\nabla u{\|}_{L^{2}{\left(\Omega_{i}^{\varepsilon }\right)}^{d}}^{2}\right).$$

In particular, repeating the arguments in the proof of Lemma 2, the trace inequality in periodic domains can be shown:

(26) $$\|u-{\left\langle T_{\varepsilon }u\right\rangle }_{Y_{i}}{\|}_{L^{2}(\partial \Omega_{i}^{\varepsilon })}^{2}⩽\varepsilon K_{\text{tr}}(1+K_{\;P})\|\nabla u{\|}_{L^{2}{\left(\Omega_{i}^{\varepsilon }\right)}^{d}}^{2}.$$

Lemma 3 (Uniform extension in connected periodic domains) For $u(x)\in H^{1}(\Omega_{i}^{\varepsilon })$, there exists a continuous extension $ũ\in H^{1}(\Omega )$ from the connected set $\Omega_{i}^{\varepsilon }$ to Ω such that ũ=u in $\Omega_{i}^{\varepsilon }$ and

(27) $$\|ũ{\|}_{L^{2}(\Omega )}^{2}⩽K_{e}\|u{\|}_{L^{2}(\Omega_{i}^{\varepsilon })}^{2},\;\|\nabla ũ{\|}_{L^{2}{\left(\Omega \right)}^{d}}^{2}⩽K_{e}\|\nabla u{\|}_{L^{2}{\left(\Omega_{i}^{\varepsilon }\right)}^{d}}^{2},\;K_{e}>0.$$

If *u*=0 on $\partial \Omega_{i}^{\varepsilon }\cap \partial \Omega$, then $ũ\in H_{0}^{1}(\Omega )$ exists satisfying (27).

Indeed, the assertion holds in accordance with previous studies,^3^, ^4^, ^45^, chapter 4 and the zero trace at the boundary *∂*Ω is argumented in Höpker.^46^, theorem 3.5

□

Below, we recall the auxiliary result from Fellner and Kovtunenko^20^, lemma 2 and Kovtunenko and Zubkova.^21^, lemma 4.1

Lemma 4 (Asymptotic restriction from Ω to $\Omega_{i}^{\varepsilon }$) For given functions *u*,*v*∈*H* ^1^(Ω) (which have no jumps across the interface Γ^*ε*^), the asymptotic estimate

(28) $$\left|\int_{\Omega_{i}^{\varepsilon }}uv\;dx-\frac{\left|Y_{i}\right|}{\left|Y\right|}\int_{\Omega }uv\;dx\right|⩽\varepsilon K_{r}\|u{\|}_{H^{1}(\Omega )}\|v{\|}_{H^{1}(\Omega )},\;K_{r}>0,$$

holds as *ε*→0 for *i*=1,2.

Based on the geometric assumptions (D1) to (D4), we define the space of periodic functions in the cells *Y*_*i*_ by

(29) $$H_{\#}^{1}(Y_{i}):=\{\varphi \in H^{1}(Y_{i}):\;\varphi (y){|}_{y_{j}=0}=\varphi (y){|}_{y_{j}=1},\;j=1,\text{⋯},d,\;\text{for}\;y\in \partial Y_{i}\cap \partial Y\}.$$

We set the standard cell problem determining $N^{i}=(N_{1}^{i},\text{⋯},N_{d}^{i})(y)$, *i*=1,2, from

(30a) $${\text{div}}_{y}\left(A_{i}(\partial_{y}N^{i}+I)\right)=0\;\text{in}\;Y_{i},$$

(30b) $$A_{i}(\partial_{y}N^{i}+I)n_{i}=0\;\text{on}\;\Gamma ,$$

(30c) $$(\partial_{y}N^{i}+I)A_{i}{|}_{y_{k}=0}={\left.(\partial_{y}N^{i}+I)A_{i}\right|}_{y_{k}=1},\;N^{i}{|}_{y_{k}=0}=N^{i}{|}_{y_{k}=1}\;\text{for}\;k=1,\text{⋯},d,$$

where the last line in (30c) implies that $N_{k}^{i}\in H_{\#}^{1}(Y_{i})$ for *i*=1,2 and *k*=1,…,*d*. In (30), the notation $\partial_{y}N^{i}(y)\in R^{d\times d}$ for *y*∈*Y*_*i*_ stands for the matrix of derivatives with entries ${\left(\partial_{y}N^{i}(y)\right)}_{kl}=\frac{\partial N_{k}^{i}}{\partial y_{l}}$, *k*,*l*=1,…,*d*, and $I\in R^{d\times d}$ denotes the identity matrix. The system (30) admits the weak formulation: find vector‐functions $N^{i}\in H_{\#}^{1}{\left(Y_{i}\right)}^{d}$ such that

(31) $$\int_{Y_{i}}A_{i}(\partial_{y}N^{i}+I)\nabla_{y}\varphi \;dy=0,$$

for all test functions $\varphi \in H_{\#}^{1}(Y_{i})$. A solution of (31) exists, and it is defined up to a constant in *Y*_*i*_.

Based on the solution *N* ^*i*^ of the cell problem (31), the diffusivity matrices *A*_*i*_ admit the following asymptotic representation formulated in the lemma below; see Fellner and Kovtunenko^20^ and Kovtunenko and Zubkova.^21^

Lemma 5 (Asymptotic formula for periodic diffusivity matrices)

(i) For the solution *N* ^*i*^ of the cell problem (31), the following representation holds:
  (32) $$A_{i}(y)(\partial_{y}N^{i}(y)+I)=A_{i}^{0}+B_{i}(y),$$
  with $A_{i}^{0}\in R_{\text{sym}}^{d\times d}$ given by the averaging
  (33) $$A_{i}^{0}:={\left\langle A_{i}(\partial_{y}N^{i}+I)\right\rangle }_{Y_{i}},$$
  and it is a symmetric *d*‐by‐*d* matrix:
  (34) $$\text{There exists}\;{\underline{a}}^{0}⩾0\;\text{such that}\;\xi^{⊤}A^{0}\;\xi ⩾{\underline{a}}^{0}|\xi {|}^{2}\;\text{for}\;\xi \in R^{d}.$$
  The *d*‐by‐*d* matrix *B*_*i*_(*y*) is periodic and has the following divergence form in the cell *Y*_*i*_:
  $${\left(B_{i}\right)}_{kl}=\sum_{m=1}^{d}b_{klm,m}^{\left(i\right)},\;k,l=1,\text{⋯},d,\;\text{where}\;b_{klm,m}^{\left(i\right)}=\frac{\partial b_{klm}^{\left(i\right)}}{\partial y_{m}}.$$
  Its components $b_{klm}^{\left(i\right)}$ are skew‐symmetric:
  $$b_{klm}^{\left(i\right)}+b_{kml}^{\left(i\right)}=0,\;k,l,m=1,\text{⋯},d,$$
  the matrix *B*_*i*_ is divergence‐free:
  $$\sum_{l,m=1}^{d}b_{klm,lm}^{\left(i\right)}=0\;\text{with}\;b_{klm,lm}^{\left(i\right)}=\frac{\partial^{2}b_{klm}^{\left(i\right)}}{\partial y_{l}\partial y_{m}},$$
  and the average ${\left\langle B_{i}\right\rangle }_{Y_{i}}=0$. At the interface, the condition holds:
  (35) $$(A_{i}^{0}+B_{i})n_{i}=0\;\text{on}\;\Gamma .$$
(ii) Assume that *N* ^*i*^∈*W* ^1,*∞*^(*Y*_*i*_)^*d*^. For varying function $v_{i}\in V_{i}^{\varepsilon }$ and fixed $u_{i}^{0}\in L^{2}(0,T;H^{3}(\Omega ))$, the following integral form corresponding to the averaged equation (50):
  (36) $$I_{A_{i}^{0}}:=\int_{\Omega_{i}^{\varepsilon }}A_{i}^{0}\nabla u_{i}^{0}\cdot \nabla v_{i}\;dx-\int_{\Gamma^{\varepsilon }}A_{i}^{0}\nabla u_{i}^{0}\cdot n_{i}v_{i}\;d\sigma_{x}$$
  with the help of the corrector $u_{i}^{1}:=u_{i}^{0}+\varepsilon (T_{\varepsilon }^{-1}N^{i})\cdot \nabla u_{i}^{0}$ is approximated as follows:
  (37) $$\begin{array}{ll} {\text{Err}}_{0}(v_{i},\varepsilon ):=\int_{0}^{T}\left(I_{A_{i}^{0}}-\int_{\Omega_{i}^{\varepsilon }}A_{i}^{\varepsilon }\nabla u_{i}^{1}\cdot \nabla v_{i}\;dx\right)\;dt, & \\ |{\text{Err}}_{0}(v_{i},\varepsilon )|⩽\varepsilon K\|A_{i}{\|}_{L^{\infty }(Y_{i})}\left(\|N^{i}{\|}_{L^{\infty }{\left(Y_{i}\right)}^{d}}+\|\partial_{y}N^{i}{\|}_{L^{\infty }{\left(Y_{i}\right)}^{d\times d}}+1\right)\|u_{i}^{0}{\|}_{L^{2}(0,T;H^{3}(\Omega_{i}^{\varepsilon }))}\|v_{i}{\|}_{L^{2}(0,T;H^{1}(\Omega_{i}^{\varepsilon }))},\;K>0. & \end{array}$$

(i) For the vector‐valued solution *N*_*i*_ of (31), the representation (32) follows from the Helmholtz theorem; see Zhikov et al.^36^, section 1.1 The interface condition (35) is obtained after substitution of (32) into (30b).
(ii) Let $v_{i}\in V_{i}^{\varepsilon }$ and $u_{i}^{0}\in L^{2}(0,T;H^{3}(\Omega ))$ be given. To prove (37), we rewrite $I_{A_{i}^{0}}$ in (36) in virtue of the integration rules from Lemma 1 in the microvariable *y*:
  (38) $$I_{A_{i}^{0}}=\frac{1}{\varepsilon^{2}|Y|}\left\{\int_{\Omega \times Y_{i}}(T_{\varepsilon }A_{i}^{0})\nabla_{y}(T_{\varepsilon }u_{i}^{0})\cdot \nabla_{y}(T_{\varepsilon }v_{i})\;dx\;dy-\int_{\Omega \times \Gamma }(T_{\varepsilon }A_{i}^{0})\nabla_{y}(T_{\varepsilon }u_{i}^{0})\cdot n_{i}(T_{\varepsilon }v_{i})\;dx\;d\sigma_{y}\right\}.$$

For the constant matrix, the identity $A_{i}^{0}=T_{\varepsilon }A_{i}^{0}$ holds. Then, expressing $A_{i}^{0}$ from (32), using the product rule

$$\partial_{y}N^{i}\nabla_{y}(T_{\varepsilon }u_{i}^{0})=\nabla_{y}(N^{i}\cdot \nabla_{y}(T_{\varepsilon }u_{i}^{0}))-\partial_{y}(\nabla_{y}(T_{\varepsilon }u_{i}^{0}))N^{i},$$

the chain rule $\varepsilon T_{\varepsilon }(\nabla u_{i}^{0})=\nabla_{y}(T_{\varepsilon }u_{i}^{0})$, and the notation of the corrector $u_{i}^{1}:=u_{i}^{0}+\varepsilon (T_{\varepsilon }^{-1}N^{i})\cdot \nabla u_{i}^{0}$, we rearrange the following terms:

$$(T_{\varepsilon }A_{i}^{0})\nabla_{y}(T_{\varepsilon }u_{i}^{0})=(A_{i}+A_{i}(\partial_{y}N^{i})-B_{i})\nabla_{y}(T_{\varepsilon }u_{i}^{0})=A_{i}\nabla_{y}(T_{\varepsilon }u_{i}^{1})-A_{i}\partial_{y}(\nabla_{y}(T_{\varepsilon }u_{i}^{0}))N^{i}-B_{i}\nabla_{y}(T_{\varepsilon }u_{i}^{0}).$$

Taking into account this formula, $I_{A_{i}^{0}}$ is performed equivalently by

(39) $$\begin{array}{ll} I_{A_{i}^{0}} & =\frac{1}{\varepsilon^{2}|Y|}\left\{\int_{\Omega \times Y_{i}}\left[A_{i}\nabla_{y}(T_{\varepsilon }u_{i}^{1}))\cdot \nabla_{y}(T_{\varepsilon }v_{i})-A_{i}\partial_{y}(\nabla_{y}(T_{\varepsilon }u_{i}^{0}))N^{i}\cdot \nabla_{y}(T_{\varepsilon }v_{i})\right]\;dx\;dy\right.\\ & \;\left.-\int_{\Omega \times \Gamma }A_{i}^{0}\nabla_{y}(T_{\varepsilon }u_{i}^{0})\cdot n_{i}(T_{\varepsilon }v_{i})\;dx\;d\sigma_{y}\right\}+I_{B_{i}}, \end{array}$$

with the integral $I_{B_{i}}$ is written component‐wisely as follows:

$$I_{B_{i}}:=-\frac{1}{\varepsilon^{2}|Y|}\int_{\Omega \times Y_{i}}B_{i}\nabla_{y}(T_{\varepsilon }u_{i}^{0})\cdot \nabla_{y}(T_{\varepsilon }v_{i})\;dx\;dy=-\frac{1}{\varepsilon^{2}|Y|}\int_{\Omega \times Y_{i}}\sum_{k,l,m=1}^{d}b_{klm,m}^{\left(i\right)}{\left(T_{\varepsilon }u_{i}^{0}\right)}_{,k}{\left(T_{\varepsilon }v_{i}\right)}_{,l}\;dx\;dy.$$

Recalling the definition of *B*_*i*_ and the fact that it is divergence‐free, the term $I_{B_{i}}$ is integrated by parts as follows:

(40) $$I_{B_{i}}=\frac{1}{\varepsilon^{2}|Y|}\int_{\Omega \times Y_{i}}\sum_{k,l,m=1}^{d}b_{klm,m}^{\left(i\right)}{\left(T_{\varepsilon }u_{i}^{0}\right)}_{,kl}(T_{\varepsilon }v_{i})\;dx\;dy-\frac{1}{\varepsilon^{2}|Y|}\int_{\Omega \times \partial Y_{i}}B_{i}\nabla_{y}(T_{\varepsilon }u_{i}^{0})\cdot n_{i}(T_{\varepsilon }v_{i})\;dx\;d\sigma_{y}.$$

After substitution of (40) in (39), the integral over Γ disappears due to the interface condition (35). The integral over *∂Y*_*i*_∖Γ vanishes after rewriting the integral again in macrovariables because of *v*_*i*_=0 on $\partial \Omega_{i}^{\varepsilon }\cap \partial \Omega$ and because jumps across the cell boundary of *v*_*i*_ and $\nabla u_{i}^{0}$ are zero (by assumed *H* ^3^‐, hence, *C* ^1^‐smoothness of $u_{i}^{0}$), while *B*_*i*_ is periodic.

The integral over Ω×*Y*_*i*_ in (40) can be rewritten using the zero average ${\left\langle B_{i}\right\rangle }_{Y_{i}}=0$ as follows:

$$\frac{1}{\varepsilon^{2}|Y|}\int_{\Omega \times Y_{i}}\sum_{k,l,m=1}^{d}b_{klm,m}^{\left(i\right)}{\left(T_{\varepsilon }u_{i}^{0}\right)}_{,kl}(T_{\varepsilon }v_{i})\;dx\;dy=I_{1}^{i}+I_{2}^{i},$$

where

$$I_{1}^{i}:=\frac{1}{\varepsilon^{2}|Y|}\int_{\Omega \times Y_{i}}\sum_{k,l,m=1}^{d}b_{klm,m}^{\left(i\right)}{\left(T_{\varepsilon }u_{i}^{0}\right)}_{,kl}(T_{\varepsilon }v_{i}-{\left\langle T_{\varepsilon }v_{i}\right\rangle }_{Y_{i}})\;dx\;dy,$$

$$I_{2}^{i}:=\frac{1}{\varepsilon^{2}|Y|}\int_{\Omega \times Y_{i}}{\left\langle T_{\varepsilon }v_{i}\right\rangle }_{Y_{i}}\sum_{k,l,m=1}^{d}b_{klm,m}^{\left(i\right)}[{\left(T_{\varepsilon }u_{i}^{0}\right)}_{,kl}-{\left\langle {\left(T_{\varepsilon }u_{i}^{0}\right)}_{,kl}\right\rangle }_{Y_{i}}]\;dx\;dy.$$

We rewrite $I_{1}^{i}$ and $I_{2}^{i}$ in the macrovariable *x* in all local cells using the integration rules (20) and (21) and then apply to the result the Cauchy‐Schwarz inequality and the Poincaré inequality (23).

Below, the indices *k*,*l*,*m* will refer to both *x* as well as *y* coordinates. We are starting from

$$I_{1}^{i}=\frac{1}{\varepsilon^{2}|Y|}\int_{\Omega \times Y_{i}}\sum_{k,l,m=1}^{d}T_{\varepsilon }(T_{\varepsilon }^{-1}b_{klm,m}^{\left(i\right)}){\left(T_{\varepsilon }u_{i}^{0}\right)}_{,kl}T_{\varepsilon }(v_{i}-{\left\langle T_{\varepsilon }v_{i}\right\rangle }_{Y_{i}})\;dx\;dy=\int_{\Omega_{i}^{\varepsilon }}\sum_{k,l,m=1}^{d}\varepsilon {\left(T_{\varepsilon }^{-1}b_{klm}^{\left(i\right)}\right)}_{,m}u_{i,kl}^{0}(v_{i}-{\left\langle v_{i}\right\rangle }_{Y_{i}^{\lambda }})\;dx,$$

where it is for all $x\in \Omega_{i}^{\varepsilon }$:

$${\left\langle T_{\varepsilon }v_{i}\right\rangle }_{Y_{i}}(x)=\frac{1}{\left|Y_{i}\right|}\int_{Y_{i}}v_{i}\left(\varepsilon \left\lfloor \frac{x}{\varepsilon }\right\rfloor +\varepsilon z\right)\;dz=\frac{1}{\left|\varepsilon (\lambda +Y_{i})\right|}\int_{\varepsilon (\lambda +Y_{i})}v_{i}\left(z\right)\;dz={\left\langle v_{i}\right\rangle }_{Y_{i}^{\lambda }}(x)$$

with $\lambda =\lfloor \frac{x}{\varepsilon }\rfloor$. First, there are some constants $0<K_{1}⩽K_{2}$ such that

(41) $$\begin{array}{ll} \left|I_{1}^{i}\right| & =\left|\sum_{\lambda \in I_{\varepsilon }}\int_{Y_{i}^{\lambda }}\sum_{k,l,m=1}^{d}(\varepsilon T_{\varepsilon }^{-1}b_{klm,m}^{\left(i\right)})u_{i,kl}^{0}(v_{i}-{\left\langle v_{i}\right\rangle }_{Y_{i}^{\lambda }})\;dx\right|\\ & ⩽K_{1}\|B_{i}{\|}_{L^{\infty }{\left(Y_{i}\right)}^{d\times d}}\|u_{i}^{0}{\|}_{H^{2}(\Omega_{i}^{\varepsilon })}\varepsilon \|\nabla v_{i}{\|}_{L^{2}{\left(\Omega_{i}^{\varepsilon }\right)}^{d}}⩽\varepsilon K_{2}(\|A_{i}{\|}_{L^{\infty }{\left(Y_{i}\right)}^{d\times d}}\|\partial_{y}N^{i}{\|}_{L^{\infty }{\left(Y_{i}\right)}^{d\times d}}+1)\|u_{i}^{0}{\|}_{H^{2}(\Omega_{i}^{\varepsilon })}\|\nabla v_{i}{\|}_{L^{2}{\left(\Omega_{i}^{\varepsilon }\right)}^{d}}. \end{array}$$

Similarly, there exists *K*_3_>0 such that

(42) $$|I_{2}^{i}|⩽K_{3}(\|A_{i}{\|}_{L^{\infty }{\left(Y_{i}\right)}^{d\times d}}\|\partial_{y}N^{i}{\|}_{L^{\infty }{\left(Y_{i}\right)}^{d\times d}}+1)\sum_{k,l=1}^{d}\varepsilon \|\nabla (u_{i,kl}^{0}){\|}_{L^{2}{\left(\Omega_{i}^{\varepsilon }\right)}^{d}}\|v_{i}{\|}_{L^{2}(\Omega_{i}^{\varepsilon })}.$$

We substitute in (39) the expression of $I_{B_{1}}$ from (40) and use (35), such that

(43) $$I_{A_{i}^{0}}-\frac{1}{\varepsilon^{2}|Y|}\int_{\Omega \times Y_{i}}A_{i}\nabla_{y}(T_{\varepsilon }u_{i}^{1})\cdot \nabla_{y}(T_{\varepsilon }v_{i})\;dx\;dy=\frac{1}{\varepsilon^{2}|Y|}\int_{\Omega \times Y_{i}}A_{i}\partial_{y}(\nabla_{y}(T^{\varepsilon }u_{i}^{0}))N^{i}\cdot \nabla_{y}(T_{\varepsilon }v_{i})\;dx\;dy+I_{1}^{i}+I_{2}^{i}.$$

Rewriting the integrals in microvariables with the help of the integration rules (20) and (21), the following estimate takes place with *K*_4_>0:

(44) $$\left|I_{A_{i}^{0}}-\int_{\Omega_{i}^{\varepsilon }}A_{i}^{\varepsilon }\nabla u_{i}^{1}\cdot \nabla v_{i}\;dx\right|⩽|I_{1}^{i}|+|I_{2}^{i}|+\varepsilon K_{4}\|A_{i}{\|}_{L^{\infty }{\left(Y_{i}\right)}^{d\times d}}\|N^{i}{\|}_{L^{\infty }{\left(Y_{i}\right)}^{d}}\|u_{i}^{0}{\|}_{H^{2}(\Omega_{i}^{\varepsilon })}\|\nabla v_{i}{\|}_{L^{2}{\left(\Omega_{i}^{\varepsilon }\right)}^{d}}.$$

Using the estimates (41) and (42), from (44) after integration over time, it follows (37) that proves the assertion of Lemma 5.

□

With these preliminaries, in the next section, we homogenize the nonlinear transmission problem (8) as *ε*→0.

## 6 THE MAIN HOMOGENIZATION RESULT

We state the averaged bidomain diffusion problem determining the functions $u_{i}^{0}(t,x)$, *i*=1,2, in the time‐space domain (0,*T*)×Ω from

(45a) $$\partial_{t}u_{i}^{0}-\text{div}(A_{i}^{0}\nabla u_{i}^{0})=\frac{\left|\Gamma \right|}{\left|Y_{i}\right|}g_{i}(u_{1}^{0},u_{2}^{0})\;\text{in}\;\Omega ,$$

(45b) $$\;u_{i}^{0}=0\;\text{on}\;\partial \Omega ,$$

(45c) $$\;u_{i}^{0}=u_{i}^{\text{in}}\;\text{as}\;t=0,$$

where the effective matrices $A_{i}^{0}$ are defined in (33). It implies the variational formulation: find $u_{i}^{0}\in U^{0}$ in the space

$$U^{0}=\{u\in L^{\infty }(0,T;L^{2}(\Omega ))\cap L^{2}(0,T;H^{1}(\Omega )):\;\partial_{t}u\in L^{2}(0,T;H^{1}{\left(\Omega \right)}^{*}),\;u=0\;\text{on}\;\partial \Omega \},$$

such that it satisfies the initial condition (45c) and the following nonlinear equation:

(46) $$\int_{0}^{T}\left({\left\langle \partial_{t}u_{i}^{0},v\right\rangle }_{\Omega }+\int_{\Omega }\left(A_{i}^{0}\nabla u_{i}^{0}\cdot \nabla v-\frac{\left|\Gamma \right|}{\left|Y_{i}\right|}g_{i}(u_{1}^{0},u_{2}^{0})v\right)\;dx\right)\;dt=0,$$

for all text functions $v\in V^{0}:=L^{2}(0,T;H_{0}^{1}(\Omega ))$. In (46), the notation ⟨·,·⟩_Ω_ implies the duality between *H* ^1^(Ω) and its topologically dual space *H* ^1^(Ω)^∗^.

The solvability of (46) can be obtained in the same way as for (8) due to the uniform boundedness (6) and the continuity (7) of the nonlinear term *g*_*i*_. Moreover, the a priori estimate like (9) holds (for *i*=1,2):

$$\|u_{i}^{0}{\|}_{U^{0}}^{2}⩽C_{1}\|u_{i}^{\text{in}}{\|}_{L^{2}(\Omega )}^{2}+C_{2}K_{g}^{2}+C_{3}.$$

In Theorem 2, we need smoothness of the macroscopic solution and the uniform boundedness of *N* ^*i*^ and of its gradient in order to prove the residual error estimate, which is a standard assumption for cell problems; see, ie, Zhikov et al.^36^, section 5.6, theorem 5.10 These assumptions might be weekend just to get a two‐scale convergence to the homogenized problem.

Theorem 2 (Residual error estimate) Let the cell problem (31) obey the Lipschitz continuous solution *N* ^*i*^∈*W* ^1,*∞*^(*Y*_*i*_), and the macroscopic solution be such that $u_{i}^{0}\in L^{2}(0,T;H^{3}(\Omega ))\cap L^{\infty }(0,T;H^{1}(\Omega ))$, $\partial_{t}(\nabla u_{i}^{0})\in L^{2}{\left(0,T;L^{2}(\Omega_{i}^{\varepsilon })\right)}^{d}$, *i*=1,2. Then the solution $u_{i}^{\varepsilon }$ of the inhomogeneous problem (8) and the first‐order corrector to the solution $u_{i}^{0}$ of the averaged problem (46) given by

(47) $$u_{i}^{1}=u_{i}^{0}+\varepsilon (T_{\varepsilon }^{-1}{Ñ}^{i})\cdot \nabla u_{i}^{0}\;\text{in}\;\Omega ,$$

where ${Ñ}^{i}\in W^{1,\infty }(Y)$ is a periodic extension of *N* ^*i*^ to *Y*, satisfy the residual error estimate:

(48) $$\|u_{i}^{\varepsilon }-u_{i}^{1}{\|}_{U_{i}^{\varepsilon }}^{2}⩽{\text{Err}}_{12}(\varepsilon )=O(\varepsilon ),$$

where Err_12_ is determined in (66).

We start with derivation of an asymptotic equation for the difference $u_{i}^{\varepsilon }-u_{i}^{1}$ (see (51)). Multiplying the diffusion equation (45a) with a test function $v_{i}\in V_{i}^{\varepsilon }$, integrating it over $(0,T)\times \Omega_{i}^{\varepsilon }$, it follows the variational equation in two subdomains for *i*=1,2:

(49) $$\int_{0}^{T}\left({\left\langle \partial_{t}u_{i}^{0},v_{i}\right\rangle }_{\Omega_{i}^{\varepsilon }}-\int_{\Omega_{i}^{\varepsilon }}\left(\text{div}(A_{i}^{0}\nabla u_{i}^{0})+\frac{\left|\Gamma \right|}{\left|Y_{i}\right|}g_{i}(u_{1}^{0},u_{2}^{0})\right)v_{i}\;dx\right)\;dt=0.$$

The integration by parts in (49) due to the Dirichlet condition (45b) leads to

(50) $$\int_{0}^{T}\left({\left\langle \partial_{t}u_{i}^{0},v_{i}\right\rangle }_{\Omega_{i}^{\varepsilon }}+\int_{\Omega_{i}^{\varepsilon }}A_{i}^{0}\nabla u_{i}^{0}\cdot \nabla v_{i}\;dx-\int_{\Gamma^{\varepsilon }}A_{i}^{0}\nabla u_{i}^{0}\cdot n_{i}v_{i}\;d\sigma_{x}\right)\;dt=\int_{0}^{T}\int_{\Omega_{i}^{\varepsilon }}\frac{\left|\Gamma \right|}{\left|Y_{i}\right|}g_{i}(u_{1}^{0},u_{2}^{0})v_{i}\;dx\;dt.$$

We choose $v\in V^{0}$ and $v_{i}\in V_{i}^{\varepsilon }$. With a special choice of *v*_*i*_, it can be equal to *v*. For test functions $v_{i}=v\in V^{0}\subset V_{i}^{\varepsilon }$, *i*=1,2, we subtract (50) from the inhomogeneous equation (8):

$$\begin{array}{ccc} & \int_{0}^{T}\left({\left\langle \partial_{t}(u_{i}^{\varepsilon }-u_{i}^{0}),v\right\rangle }_{\Omega_{i}^{\varepsilon }}+\int_{\Omega_{i}^{\varepsilon }}\left(A_{i}^{\varepsilon }\nabla u_{i}^{\varepsilon }-A_{i}^{0}\nabla u_{i}^{0}\right)\cdot \nabla v\;dx+\int_{\Gamma^{\varepsilon }}A_{i}^{0}\nabla u_{i}^{0}\cdot n_{i}v\;d\sigma_{x}\right)\;dt & \\ & =\int_{0}^{T}\left(\int_{\Gamma^{\varepsilon }}\varepsilon g_{i}(u_{1}^{\varepsilon },u_{2}^{\varepsilon })v\;d\sigma_{x}-\int_{\Omega_{i}^{\varepsilon }}\frac{\left|\Gamma \right|}{\left|Y_{i}\right|}g_{i}(u_{1}^{0},u_{2}^{0})v\;dx\right)\;dt \end{array}$$

and gather the terms as follows:

(51) $$\int_{0}^{T}\left({\left\langle \partial_{t}(u_{i}^{\varepsilon }-u_{i}^{1}),v\right\rangle }_{\Omega_{i}^{\varepsilon }}+\int_{\Omega_{i}^{\varepsilon }}A_{i}^{\varepsilon }\nabla (u_{i}^{\varepsilon }-u_{i}^{1})\cdot \nabla v\;dx\right)\;dt-I_{i}(v)=\sum_{k=0}^{3}{\text{Err}}_{k}(v,\varepsilon ),$$

where the following notation was used

(52) $$I_{i}(v):=\int_{0}^{T}\left(\int_{\Gamma^{\varepsilon }}\varepsilon g_{i}(u_{1}^{\varepsilon },u_{2}^{\varepsilon })v\;d\sigma_{x}-\frac{\left|\Gamma \right|}{\left|Y\right|}\int_{\Omega }g_{i}(u_{1}^{1},u_{2}^{1})v\;dx\right)\;dt.$$

Err_0_ is given by the formula (37) from Lemma 5, and other residual error functions Err_*k*_, *k*=1,2,3, in the right‐hand side of (51) will be introduced and estimated next.

We use the Cauchy‐Schwarz inequality and the expansion of the time‐derivative of the corrector $\partial_{t}u_{i}^{1}=\partial_{t}[u_{i}^{0}+\varepsilon (T_{\varepsilon }^{-1}N^{i})\cdot \nabla u_{i}^{0}]$ implying that

(53) $$\begin{array}{ll} {\text{Err}}_{1}(v,\varepsilon ) & :=-\int_{0}^{T}{\left\langle \partial_{t}(u_{i}^{1}-u_{i}^{0}),v\right\rangle }_{\Omega_{i}^{\varepsilon }}\;dt,\\ \left|{\text{Err}}_{1}(v,\varepsilon )\right| & ⩽\|\partial_{t}u_{i}^{1}-\partial_{t}u_{i}^{0}{\|}_{L^{2}(0,T;H^{1}{\left(\Omega_{i}^{\varepsilon }\right)}^{*})}\|v{\|}_{L^{2}(0,T;H^{1}(\Omega_{i}^{\varepsilon }))}⩽\varepsilon \|N^{i}{\|}_{L^{\infty }{\left(Y_{i}\right)}^{d}}\|\partial_{t}(\nabla u_{i}^{0}){\|}_{L^{2}{\left(0,T;H^{1}{\left(\Omega_{i}^{\varepsilon }\right)}^{*}\right)}^{d}}\|v{\|}_{L^{2}(0,T;H^{1}(\Omega_{i}^{\varepsilon }))}. \end{array}$$

Applying to $g_{i}(u_{1}^{0},u_{2}^{0})v$ the restriction operator from Lemma 4, then using the boundedness (6) and the Lipschitz continuity (7) for *g*_*i*_ leads to

(54) $$\begin{array}{ll} {\text{Err}}_{2}(v,\varepsilon ) & :=-\int_{0}^{T}\left(\frac{\left|\Gamma \right|}{\left|Y_{i}\right|}\int_{\Omega_{i}^{\varepsilon }}g_{i}(u_{1}^{0},u_{2}^{0})v\;dx-\frac{\left|\Gamma \right|}{\left|Y\right|}\int_{\Omega }g_{i}(u_{1}^{0},u_{2}^{0})v\;dx\right)\;dt\\ \left|{\text{Err}}_{2}(v,\varepsilon )\right| & ⩽\varepsilon K_{6}K_{g}\|v{\|}_{L^{2}(0,T;H^{1}(\Omega ))},\;K_{6}=\frac{K_{r}|\Gamma |}{\left|Y_{i}\right|}\sqrt{T|\Omega |}, \end{array}$$

and the further error function (with *K*_7_=|Γ|*L*_g_)

(55) $$\begin{array}{ll} {\text{Err}}_{3}(v,\varepsilon ) & :=\frac{\left|\Gamma \right|}{\left|Y\right|}\int_{0}^{T}\int_{\Omega }(g_{i}(u_{1}^{1},u_{2}^{1})-g_{i}(u_{1}^{0},u_{2}^{0}))v\;dx\;dt,\\ \left|{\text{Err}}_{3}(v,\varepsilon )\right| & ⩽\frac{|\Gamma |L_{g}}{\left|Y\right|}\sum_{j=1}^{2}\|u_{j}^{1}-u_{j}^{0}{\|}_{L^{2}(0,T;L^{2}(\Omega ))}\|v{\|}_{L^{2}(0,T;L^{2}(\Omega ))}⩽\varepsilon K_{7}\sum_{j=1}^{2}\|{Ñ}^{j}{\|}_{L^{\infty }{\left(Y\right)}^{d}}\|\nabla u_{j}^{0}{\|}_{L^{2}{\left(0,T;L^{2}(\Omega )\right)}^{d}}\|v{\|}_{L^{2}(0,T;L^{2}(\Omega ))}. \end{array}$$

In the following, we aim at substitution of *v* by piecewise constant average $\left\langle T_{\varepsilon }v\rangle (x):={\left\langle T_{\varepsilon }v\right\rangle }_{Y_{j}}(x\right)$ for $x\in \Omega_{j}^{\varepsilon }$, *j*=1,2. For this task, we decompose *I*_*i*_ in (52) as follows:

$$I_{i}(v)=J_{i}(\langle T_{\varepsilon }v\rangle )+{\text{Err}}_{4}(v,\varepsilon ),$$

with the terms defined as

$$J_{i}(\langle T_{\varepsilon }v\rangle ):=\frac{1}{\left|Y\right|}\int_{0}^{T}\int_{\Omega \times \Gamma }\left(g_{i}(T_{\varepsilon }u_{1}^{\varepsilon },T_{\varepsilon }u_{2}^{\varepsilon })-g_{i}(u_{1}^{1},u_{2}^{1})\right)\langle T_{\varepsilon }v\rangle \;dx\;d\sigma_{y}\;dt,$$

$$\begin{array}{ccc} {\text{Err}}_{4}(v,\varepsilon ) & :=\int_{0}^{T}\left(\int_{\Gamma^{\varepsilon }}\varepsilon g_{i}(u_{1}^{\varepsilon },u_{2}^{\varepsilon })v\;d\sigma_{x}-\frac{1}{\left|Y\right|}\int_{\Omega \times \Gamma }g_{i}(T_{\varepsilon }u_{1}^{\varepsilon },T_{\varepsilon }u_{2}^{\varepsilon })\langle T_{\varepsilon }v\rangle \;dx\;d\sigma_{y}\right. & \\ & \;\left.-\frac{\left|\Gamma \right|}{\left|Y\right|}\int_{\Omega }g_{i}(u_{1}^{1},u_{2}^{1})v\;dx+\frac{1}{\left|Y\right|}\int_{\Omega \times \Gamma }g_{1}(u_{1}^{1},u_{2}^{1})\langle T_{\varepsilon }v\rangle \;dx\;d\sigma_{y}\right)\;dt. \end{array}$$

We apply the integration rule (20b) to the first term of Err_4_ and rewrite the third term using $|\Gamma |=\int_{\Gamma }\;d\sigma_{y}$. Based on the boundedness (6) of *g*_*i*_, from the Cauchy‐Schwarz inequality, it follows the error estimate

(56) $$\begin{array}{ll} \left|{\text{Err}}_{4}(v,\varepsilon )\right| & =\frac{1}{\left|Y\right|}\left|\int_{0}^{T}\int_{\Omega \times \Gamma }g_{i}(T_{\varepsilon }u_{1}^{\varepsilon },T_{\varepsilon }u_{2}^{\varepsilon })(T_{\varepsilon }v-\langle T_{\varepsilon }v\rangle )\;dx\;d\sigma_{y}\;dt-\int_{0}^{T}\int_{\Omega \times \Gamma }g_{i}(u_{1}^{1},u_{2}^{1})(v-\langle T_{\varepsilon }v\rangle )\;dx\;d\sigma_{y}\;dt\right|\\ & ⩽\|\varepsilon g_{i}(u_{1}^{\varepsilon },u_{2}^{\varepsilon }){\|}_{L^{2}(0,T;L^{2}(\Gamma^{\varepsilon }))}\frac{1}{\sqrt{\left|Y\right|}}\|T_{\varepsilon }v-\langle T_{\varepsilon }v\rangle {\|}_{L^{2}(0,T;L^{2}(\Omega \times \Gamma ))}\\ & \;+\frac{1}{\left|Y\right|}\|g_{i}(u_{1}^{1},u_{2}^{1}){\|}_{L^{2}(0,T;L^{2}(\Omega \times \Gamma ))}\|v-\langle T_{\varepsilon }v\rangle {\|}_{L^{2}(0,T;L^{2}(\Omega \times \Gamma ))}\\ & ⩽\varepsilon K_{8}K_{g}\|\nabla v{\|}_{L^{2}{\left(0,T;L^{2}(\Omega )\right)}^{d}}, \end{array}$$

where $K_{8}=\sqrt{\varepsilon T|\Gamma^{\varepsilon }|K_{\text{tr}}(1+K_{P})}+\frac{\left|\Gamma \right|}{\left|Y\right|}\sqrt{T|\Omega |K_{P}}$. Here, we have used the Poincaré inequality (22), following the trace inequality in periodic domains (26) such that

$$\begin{array}{ccc} \int_{\Omega \times \Gamma }{\left(T_{\varepsilon }v-\langle T_{\varepsilon }v\rangle \right)}^{2}\;dx\;d\sigma_{y} & =\sum_{j=1}^{2}\int_{\Omega_{j}^{\varepsilon }\times \Gamma }{\left(T_{\varepsilon }v-{\left\langle T_{\varepsilon }v\right\rangle }_{Y_{j}}\right)}^{2}\;dx\;d\sigma_{y} & \\ & ⩽\sum_{j=1}^{2}K_{\text{tr}}\int_{\Omega_{j}^{\varepsilon }\times Y_{j}}\left({\left(T_{\varepsilon }v-{\left\langle T_{\varepsilon }v\right\rangle }_{Y_{j}}\right)}^{2}+|\nabla_{y}(T_{\varepsilon }v){|}^{2}\right)\;dx\;dy\\ & ⩽K_{\text{tr}}(1+K_{P})\sum_{j=1}^{2}\int_{\Omega_{j}^{\varepsilon }\times Y_{j}}|\nabla_{y}(T_{\varepsilon }v){|}^{2}\;dx\;dy⩽\varepsilon |Y|K_{\text{tr}}(1+K_{\;P})\int_{\Omega }|\nabla v{|}^{2}\;dx. \end{array}$$

Applying Young inequality to *J*_*i*_ implies that

$$|J_{i}(\langle T_{\varepsilon }v\rangle )|⩽\frac{1}{\left|Y\right|}\int_{0}^{T}\int_{\Omega \times \Gamma }\left(\frac{1}{2}{\left|g_{i}(T_{\varepsilon }u_{1}^{\varepsilon },T_{\varepsilon }u_{2}^{\varepsilon })-g_{i}(u_{1}^{1},u_{2}^{1})\right|}^{2}+\frac{1}{2}{\left\langle T_{\varepsilon }v\right\rangle }^{2}\right)\;dx\;d\sigma_{y}\;dt.$$

Due to the Lipschitz continuity (7) of *g*_*i*_, using the mean inequality

$$|T_{\varepsilon }u_{i}^{\varepsilon }-u_{i}^{1}{|}^{2}⩽2|T_{\varepsilon }(u_{i}^{\varepsilon }-u_{i}^{1}){|}^{2}+2|T_{\varepsilon }u_{i}^{1}-u_{i}^{1}{|}^{2},$$

application of the integration rule (21c) and the trace inequality (25) proceeds further

(57) $$\begin{array}{ll} \left|J_{i}(\langle T_{\varepsilon }v\rangle )\right| & ⩽\frac{1}{\left|Y\right|}\int_{0}^{T}\int_{\Omega \times \Gamma }\left(2L_{g}^{2}\sum_{j=1}^{2}|T_{\varepsilon }(u_{j}^{\varepsilon }-u_{j}^{1}){|}^{2}+\frac{1}{2}{\left\langle T_{\varepsilon }v\right\rangle }^{2}\right)\;dx\;d\sigma_{y}\;dt+{\text{Err}}_{5}(v,\varepsilon )\\ & =2\varepsilon L_{g}^{2}\sum_{j=1}^{2}\int_{0}^{T}\int_{\Gamma^{\varepsilon }}|u_{j}^{\varepsilon }-u_{j}^{1}{|}^{2}d\sigma_{x}\;dt+\frac{\left|\Gamma \right|}{2}\sum_{j=1}^{2}\int_{0}^{T}\int_{\Omega_{j}^{\varepsilon }}{\left\langle T_{\varepsilon }v\right\rangle }^{2}\;dx\;dt+{\text{Err}}_{5}(v,\varepsilon )\\ & ⩽2K_{\text{tr}}L_{g}^{2}\sum_{j=1}^{2}\left(\|u_{j}^{\varepsilon }-u_{j}^{1}{\|}_{L^{2}(0,T;L^{2}(\Omega_{j}^{\varepsilon }))}^{2}+\varepsilon^{2}\|\nabla (u_{j}^{\varepsilon }-u_{j}^{1}){\|}_{L^{2}{\left(0,T;L^{2}(\Omega_{j}^{\varepsilon })\right)}^{d}}^{2}\right)+\frac{\left|\Gamma \right|}{2}\sum_{j=1}^{2}\frac{1}{|Y_{j}{|}^{2}}\|v{\|}_{L^{2}(0,T;L^{2}(\Omega ))}+{\text{Err}}_{5}(v,\varepsilon ), \end{array}$$

because of (see Cioranescu et al^43^, proposition 2.17)

$$\|\langle T_{\varepsilon }v\rangle {\|}_{L^{2}(\Omega_{j}^{\varepsilon })}=\frac{\left|Y\right|}{\left|Y_{j}\right|}\|T_{\varepsilon }^{-1}v{\|}_{L^{2}(\Omega )}⩽\frac{\sqrt{\left|Y\right|}}{\left|Y_{j}\right|}\|v{\|}_{L^{2}(\Omega )},$$

where

$${\text{Err}}_{5}(v,\varepsilon ):=\frac{2L_{g}^{2}}{\left|Y\right|}\sum_{j=1}^{2}\int_{0}^{T}\int_{\Omega \times \Gamma }{\left|T_{\varepsilon }u_{j}^{1}-u_{j}^{1}\right|}^{2}\;dx\;d\sigma_{y}\;dt.$$

First, we estimate Err_5_ in (57). Since $u_{i}^{1}\in H^{1}(\Omega )$, according to Griso,^29^, formula (3.4) the auxiliary estimate for the term in Err_5_ holds:

$$\|T_{\varepsilon }u_{j}^{1}-u_{j}^{1}{\|}_{L^{2}(\Omega \times Y_{j})}^{2}⩽\varepsilon^{2}K_{c}\|\nabla u_{j}^{1}{\|}_{L^{2}{\left(\Omega \right)}^{d}}^{2},\;K_{c}>0.$$

Therefore, from the trace theorem (24) in Ω×*Y*_*j*_ and (21b), we have

$$\frac{1}{\left|Y\right|}\|T_{\varepsilon }u_{j}^{1}-u_{j}^{1}{\|}_{L^{2}(\Omega \times \Gamma )}^{2}⩽\frac{K_{\text{tr}}}{\left|Y\right|}\left(\|T_{\varepsilon }u_{j}^{1}-u_{j}^{1}{\|}_{L^{2}(\Omega \times Y_{j})}^{2}+\|\nabla_{y}(T_{\varepsilon }u_{j}^{1}){\|}_{L^{2}{\left(\Omega \times Y_{j}\right)}^{d}}^{2}\right)\varepsilon^{2}K_{u}\|\nabla u_{j}^{1}{\|}_{L^{2}{\left(\Omega \right)}^{d}}^{2},\;K_{u}:=K_{\text{tr}}\left(\frac{K_{c}}{\left|Y\right|}+1\right),$$

and the term Err_5_(*v*,*ε*) is estimated by

(58) $$0⩽{\text{Err}}_{5}(v,\varepsilon )⩽2\varepsilon^{2}L_{g}^{2}K_{u}\sum_{j=1}^{2}\|\nabla u_{j}^{1}{\|}_{L^{2}{\left(0,T;L^{2}(\Omega )\right)}^{d}}^{2}.$$

Let *η*_Ω_(*x*) be a smooth cutoff function with a compact support in Ω and equals one outside an *ε*‐neighborhood of the boundary *∂*Ω such that $|\eta_{\Omega }|⩽1$ and $\varepsilon |\nabla \eta_{\Omega }|⩽C_{\eta }$. For further use, we employ the following functions $w_{i}\in V^{0}\subset V_{i}^{\varepsilon }$ expressed equivalently in two ways as

(59) $$w_{i}:={ũ}_{i}^{\varepsilon }-u_{i}^{0}-\varepsilon (T_{\varepsilon }^{-1}{Ñ}^{i})\cdot \nabla u_{i}^{0}\eta_{\Omega }={ũ}_{i}^{\varepsilon }-u_{i}^{1}+\varepsilon (T_{\varepsilon }^{-1}{Ñ}^{i})\cdot \nabla u_{i}^{0}(1-\eta_{\Omega }),$$

where ${ũ}_{i}^{\varepsilon }\in H_{0}^{1}(\Omega )$ is the uniform extension of $u_{i}^{\varepsilon }\in U_{i}^{\varepsilon }$ according to Lemma 3.

We will derive the estimates for ${ũ}_{i}^{\varepsilon }-u_{i}^{1}$ with the help of substitution of the test function *v*=*w*_*i*_ from (59) into the expressions for Err_*k*_(*v*,*ε*), *k*=0,1,…,5. This implies the following structure of the bounds:

(60) $$|{\text{Err}}_{k}(w_{i},\varepsilon )|⩽\varepsilon \alpha_{k}U_{k},$$

where the terms are defined by means of

$$\begin{array}{ll} \alpha_{0}:=K\|A_{i}{\|}_{L^{\infty }(Y_{i})}\left(\|N^{i}{\|}_{W^{1,\infty }{\left(Y_{i}\right)}^{d}}+1\right),\; & U_{0}:=\|u_{i}^{0}{\|}_{L^{2}(0,T;H^{3}(\Omega_{i}^{\varepsilon }))}\|w_{i}{\|}_{L^{2}(0,T;H^{1}(\Omega_{i}^{\varepsilon }))},\\ \alpha_{1}:=\|N^{i}{\|}_{L^{\infty }{\left(Y_{i}\right)}^{d}},\; & U_{1}:=\|\partial_{t}(\nabla u_{i}^{0}){\|}_{L^{2}{\left(0,T;H^{1}{\left(\Omega_{i}^{\varepsilon }\right)}^{*}\right)}^{d}}\|w_{i}{\|}_{L^{2}(0,T;H^{1}(\Omega_{i}^{\varepsilon }))},\\ \alpha_{2}:=K_{6}K_{g}, & U_{2}:=\|w_{i}{\|}_{L^{2}(0,T;H^{1}(\Omega ))},\\ \alpha_{3}:=K_{7}\sum_{j=1}^{2}\|{Ñ}^{j}{\|}_{L^{\infty }{\left(Y\right)}^{d}}, & U_{3}:=\sum_{j=1}^{2}\|\nabla u_{j}^{0}{\|}_{L^{2}{\left(0,T;L^{2}(\Omega )\right)}^{d}}\|w_{j}{\|}_{L^{2}(0,T;L^{2}(\Omega ))},\\ \alpha_{4}:=K_{8}K_{g}, & U_{4}:=\|\nabla w_{i}{\|}_{L^{2}{\left(0,T;L^{2}(\Omega )\right)}^{d}},\\ \alpha_{5}:=2\varepsilon L_{g}^{2}K_{u}, & U_{5}:=\sum_{j=1}^{2}\|\nabla u_{j}^{1}{\|}_{L^{2}{\left(0,T;L^{2}(\Omega )\right)}^{d}}^{2}, \end{array}$$

According to the uniform estimate (9) in Theorem 1 and the continuous extension (27), we have

(61) $$\left\|w_{i}{\|}_{L^{2}(0,T;H^{1}(\Omega ))}^{2}⩽3K_{e}\|u_{i}^{\varepsilon }{\|}_{L^{2}(0,T;H^{1}(\Omega_{i}^{\varepsilon }))}^{2}+3\|u_{i}^{0}{\|}_{L^{2}(0,T;H^{1}(\Omega ))}^{2}+3\varepsilon \|{Ñ}_{i}{\|}_{L^{\infty }{\left(Y\right)}^{d}}\|\sqrt{\varepsilon }\nabla u_{i}^{0}\eta_{\Omega }{\|}_{L^{2}{\left(0,T;H^{1}(\Omega )\right)}^{d}}^{2}=O(1\right)$$

following that all *α*_*k*_=*O*(1) and *U*_*k*_=*O*(1) for *k*=0,1,…,5.

The asymptotic equation (51) tested with the function *v*=*w*_*i*_ from (59) leads to

(62) $$\begin{array}{ll} \frac{1}{2}\frac{d}{dt}\int_{0}^{T}\int_{\Omega_{i}^{\varepsilon }}{\left(u_{i}^{\varepsilon }-u_{i}^{1}\right)}^{2}\;dx\;dt+\int_{0}^{T}\int_{\Omega_{i}^{\varepsilon }}A_{i}^{\varepsilon }\nabla (u_{i}^{\varepsilon }-u_{i}^{1})\cdot \nabla (u_{i}^{\varepsilon }-u_{i}^{1})\;dx\;dt & \\ =J_{i}(\langle T_{\varepsilon }w_{i}\rangle )+\sum_{k=0}^{4}{\text{Err}}_{k}(w_{i},\varepsilon )+{\text{Err}}_{6}(\varepsilon )+M(u_{i}^{\varepsilon }-u_{i}^{1}) & \end{array}$$

with the following two terms:

$$\begin{array}{ccc} {\text{Err}}_{6}(\varepsilon ) & :=-\int_{0}^{T}{\left\langle \partial_{t}(u_{i}^{\varepsilon }-u_{i}^{1}),\varepsilon (T_{\varepsilon }^{-1}N_{i})\cdot \nabla u_{i}^{0}(1-\eta_{\Omega })\right\rangle }_{\Omega_{i}^{\varepsilon }}\;dt, & \\ M(u_{i}^{\varepsilon }-u_{i}^{1}) & :=-\int_{0}^{T}\int_{\Omega_{i}^{\varepsilon }}A_{i}^{\varepsilon }\nabla (u_{i}^{\varepsilon }-u_{i}^{1})\cdot \nabla [\varepsilon (T_{\varepsilon }^{-1}N^{i})\cdot \nabla u_{i}^{0}(1-\eta_{\Omega })]\;dx\;dt. \end{array}$$

We note that *M* is not an error term; in contrary, it enters with the factor −*δ*_1_ the left‐hand side of the estimate (65) following later.

Err_6_ is estimated by integration by parts with respect to time

$${\text{Err}}_{6}(\varepsilon )=\int_{0}^{T}\int_{\Omega_{i}^{\varepsilon }}(u_{i}^{\varepsilon }-u_{i}^{1})\varepsilon (T_{\varepsilon }^{-1}N_{i})\cdot \partial_{t}(\nabla u_{i}^{0})(1-\eta_{\Omega })\;dx\;dt-\int_{\Omega_{i}^{\varepsilon }}{\left.(u_{i}^{\varepsilon }-u_{i}^{1})\varepsilon (T_{\varepsilon }^{-1}N_{i})\cdot \nabla u_{i}^{0}(1-\eta_{\Omega })\;dx\right|}_{t=0}^{T},$$

after using Young inequality and the continuous embedding

(63) $$\|u_{i}^{\varepsilon }-u_{i}^{1}{\|}_{L^{2}(0,T;L^{2}(\Omega_{i}^{\varepsilon }))}^{2}⩽K_{\text{emb}}\|u_{i}^{\varepsilon }-u_{i}^{1}{\|}_{L^{\infty }(0,T;L^{2}(\Omega_{i}^{\varepsilon }))}^{2},$$

which implies that

$$|{\text{Err}}_{6}(\varepsilon )|⩽\varepsilon \alpha_{6}U_{6},$$

where

$$\begin{array}{ccc} \alpha_{6} & :=\frac{2+K_{emb}}{2}\|N^{i}{\|}_{L^{\infty }{\left(Y_{i}\right)}^{d}}, & \\ U_{6} & :=\frac{1}{2+K_{\text{emb}}}\left(\|\partial_{t}(\nabla u_{i}^{0})(1-\eta_{\Omega }){\|}_{L^{2}{\left(0,T;L^{2}(\Omega_{i}^{\varepsilon })\right)}^{d}}^{2}+2\|\nabla u_{i}^{0}(1-\eta_{\Omega }){\|}_{L^{\infty }{\left(0,T;L^{2}(\Omega_{i}^{\varepsilon })\right)}^{d}}^{2}\right)+\|u_{i}^{\varepsilon }-u_{i}^{1}{\|}_{L^{\infty }(0,T;L^{2}(\Omega_{i}^{\varepsilon }))}^{2}. \end{array}$$

The term $M(u_{i}^{\varepsilon }-u_{i}^{1})$ is evaluated by Young inequality with the weight *δ*_1_>0 and using the boundedness property of *A*_*i*_ with the upper bound *β* from (5) as

$$\begin{array}{ccc} \left|M(u_{i}^{\varepsilon }-u_{i}^{1})\right| & =\left|\int_{0}^{T}\int_{\Omega_{i}^{\varepsilon }}A_{i}^{\varepsilon }\nabla (u_{i}^{\varepsilon }-u_{i}^{1})\cdot \{T_{\varepsilon }^{-1}(\partial_{y}N^{i})\cdot \nabla u_{i}^{0}(1-\eta_{\Omega })+\varepsilon (T_{\varepsilon }^{-1}N^{i})\cdot \partial_{x}(\nabla u_{i}^{0})(1-\eta_{\Omega })\right. & \\ & \;\left.-\varepsilon (T_{\varepsilon }^{-1}N^{i})\cdot \nabla u_{i}^{0}\nabla \eta_{\Omega }\}\;dx\;dt\right|⩽\frac{3\beta \delta_{1}}{2\sqrt{3}}\|\nabla (u_{i}^{\varepsilon }-u_{i}^{1}){\|}_{L^{2}{\left(0,T;L^{2}(\Omega_{i}^{\varepsilon })\right)}^{d}}^{2}+{\text{Err}}_{7}(\varepsilon ), \end{array}$$

where

$$\begin{array}{ccc} {\text{Err}}_{7}(\varepsilon ) & :=\frac{\sqrt{3}\beta }{2\delta_{1}}\left\{\|\partial_{y}N^{i}{\|}_{L^{\infty }{\left(Y_{i}\right)}^{d\times d}}\|\nabla u_{i}^{0}(1-\eta_{\Omega }){\|}_{L^{2}{\left(0,T;L^{2}(\Omega_{i}^{\varepsilon })\right)}^{d}}^{2}\right. & \\ & \;\left.+\varepsilon^{2}\|N^{i}{\|}_{L^{\infty }{\left(Y_{i}\right)}^{d}}\|\partial_{x}(\nabla u_{i}^{0})(1-\eta_{\Omega }){\|}_{L^{2}{\left(0,T;L^{2}(\Omega_{i}^{\varepsilon })\right)}^{d\times d}}^{2}+\varepsilon^{2}\|N^{i}{\|}_{L^{\infty }{\left(Y_{i}\right)}^{d}}\|\nabla u_{i}^{0}\cdot \nabla \eta_{\Omega }{\|}_{L^{2}(0,T;L^{2}(\Omega_{i}^{\varepsilon }))}^{2}\right\}. \end{array}$$

It follows

$$|{\text{Err}}_{7}(\varepsilon )|⩽\varepsilon \alpha_{7}U_{7},$$

where

$$\begin{array}{ccc} \alpha_{7} & :=\frac{\sqrt{3}\beta }{2}\left(\|N^{i}{\|}_{L^{\infty }{\left(Y_{i}\right)}^{d}}+\|\partial_{y}N^{i}{\|}_{L^{\infty }{\left(Y_{i}\right)}^{d\times d}}\right), & \\ U_{7} & :=\frac{1}{\delta_{1}}\left(\|\nabla u_{i}^{0}(1-\eta_{\Omega }){\|}_{L^{2}{\left(0,T;L^{2}(\Omega_{i}^{\varepsilon })\right)}^{d}}^{2}+\varepsilon \|\partial_{x}(\nabla u_{i}^{0})(1-\eta_{\Omega }){\|}_{L^{2}{\left(0,T;L^{2}(\Omega_{i}^{\varepsilon })\right)}^{d\times d}}^{2}+\varepsilon \|\nabla u_{i}^{0}\cdot \nabla \eta_{\Omega }{\|}_{L^{2}(0,T;L^{2}(\Omega_{i}^{\varepsilon }))}^{2}\right)=O(1). \end{array}$$

We note that *U*_7_=*O*(1), in particular, because 1−*η*_Ω_≠0 on a *O*(*ε*)‐set using the fact that 1−*η*_Ω_≠0 on a set of measure *O*(*ε*), thus compensating ∇*η*_Ω_=*O*(*ε* ^−1^) here.

Therefore, using the inequality (57) for *J*_*i*_(⟨*T*_*ε*_ *w*_*i*_⟩) and the uniform positive definiteness (33) of *A*_*i*_ with the lower bound *α*>0, from (62), we arrive at the estimate

(64) $$\begin{array}{ll} & \left|\frac{1}{2}\int_{\Omega_{i}^{\varepsilon }}{\left(u_{i}^{\varepsilon }-u_{i}^{1}\right)}^{2}\left|{}_{t=0}^{T}\;dx+\left(\alpha -\frac{\sqrt{3}\beta \delta_{1}}{2}\right)\right.\int_{0}^{T}\int_{\Omega_{i}^{\varepsilon }}|\nabla (u_{i}^{\varepsilon }-u_{i}^{1}){|}^{2}\;dx\;dt\right|\\ & ⩽(2K_{\text{tr}}L_{g}^{2}+\alpha_{8})\sum_{i=1}^{2}\|u_{i}^{\varepsilon }-u_{i}^{1}{\|}_{L^{2}(0,T;L^{2}(\Omega_{i}^{\varepsilon }))}^{2}+2\varepsilon^{2}K_{\text{tr}}L_{g}^{2}\sum_{i=1}^{2}\|\nabla (u_{i}^{\varepsilon }-u_{i}^{1}){\|}_{L^{2}{\left(0,T;L^{2}(\Omega_{i}^{\varepsilon })\right)}^{d}}^{2}\\ & +\sum_{k=0}^{5}|{\text{Err}}_{k}(w_{i},\varepsilon )|+\sum_{k=6}^{8}|{\text{Err}}_{k}(\varepsilon )|, \end{array}$$

where $\alpha_{8}:=\frac{\left|\Gamma \right|}{2}\sum_{j=1}^{2}\frac{1}{|Y_{j}{|}^{2}}$, and

$$0⩽{\text{Err}}_{8}(\varepsilon ):=\alpha_{8}\|\varepsilon (T_{\varepsilon }^{-1}N^{i})\cdot \nabla u_{i}^{0}(1-\eta_{\Omega }){\|}_{L^{2}(0,T;L^{2}(\Omega_{i}^{\varepsilon }))}^{2}⩽\varepsilon^{2}\alpha_{8}\|N^{i}{\|}_{L^{\infty }{\left(Y_{i}\right)}^{d}}\|\nabla u_{i}^{0}{\|}_{L^{2}{\left(0,T;L^{2}(\Omega_{i}^{\varepsilon })\right)}^{d}}.$$

After summation over *i*=1,2 we rearrange the terms such that

(65) $$\begin{array}{ll} & \frac{1}{2}\sum_{i=1}^{2}\|(u_{i}^{\varepsilon }-u_{i}^{1})(T){\|}_{L^{2}(\Omega_{i}^{\varepsilon })}^{2}+\gamma \sum_{i=1}^{2}\|\nabla (u_{i}^{\varepsilon }-u_{i}^{1}){\|}_{L^{2}{\left(0,T;L^{2}(\Omega_{i}^{\varepsilon })\right)}^{d}}^{2}⩽\alpha_{10}\sum_{i=1}^{2}\|u_{i}^{\varepsilon }-u_{i}^{1}{\|}_{L^{2}(0,T;L^{2}(\Omega_{i}^{\varepsilon }))}^{2}+{\text{Err}}_{10}(\varepsilon ),\\ & {\text{Err}}_{10}(\varepsilon ):=\sum_{k=0}^{5}\sum_{i=1}^{2}|{\text{Err}}_{k}(w_{i},\varepsilon )|+2\sum_{k=6}^{9}|{\text{Err}}_{k}(\varepsilon )|, \end{array}$$

where $\gamma :=\alpha -4\varepsilon^{2}K_{\text{tr}}L_{g}^{2}-\frac{\sqrt{3}\beta \delta_{1}}{2}$, $\alpha_{10}:=2(K_{\text{tr}}L_{g}^{2}+\alpha_{8})$, and the error Err_9_ implies

$${\text{Err}}_{9}(\varepsilon ):=\frac{1}{2}\|(u_{i}^{\varepsilon }-u_{i}^{1})(0){\|}_{L^{2}(\Omega_{i}^{\varepsilon })}^{2}⩽\frac{\varepsilon }{2}\|N^{i}{\|}_{L^{\infty }{\left(Y_{i}\right)}^{d}}\|\nabla u_{i}^{0}(0){\|}_{L^{2}{\left(\Omega_{i}^{\varepsilon }\right)}^{d}}^{2}=O(\varepsilon ).$$

After taking the supremum over time, using the embedding theorem (63), we estimate the first term in the left‐hand side of (65) by the lower bound

$$\frac{1}{2}\sum_{i=1}^{2}\|(u_{i}^{\varepsilon }-u_{i}^{1})(T){\|}_{L^{2}(\Omega_{i}^{\varepsilon })}^{2}⩾\frac{1}{4K_{\text{emb}}}\sum_{i=1}^{2}\|u_{i}^{\varepsilon }-u_{i}^{1}{\|}_{L^{2}(0,T;L^{2}(\Omega_{i}^{\varepsilon }))}^{2}+\frac{1}{4}\sum_{i=1}^{2}\|u_{i}^{\varepsilon }-u_{i}^{1}{\|}_{L^{\infty }(0,T;L^{2}(\Omega_{i}^{\varepsilon }))}^{2}.$$

We continue the estimate (65) by taking *δ*_1_ small enough such that *γ*>0. Therefore, applying Grönwall inequality leads to

$$\sum_{i=1}^{2}\|(u_{i}^{\varepsilon }-u_{i}^{1})(t){\|}_{L^{2}(\Omega_{i}^{\varepsilon })}^{2}⩽{\text{Err}}_{11}(\varepsilon ),\;{\text{Err}}_{11}(\varepsilon ):=2{\text{Err}}_{10}(\varepsilon )\exp(2\alpha_{10}T).$$

As a consequence, from (65) and the embedding theorem (63), we conclude with the estimate

(66) $$\begin{array}{ll} & \sum_{i=1}^{2}\|u_{i}^{\varepsilon }-u_{i}^{1}{\|}_{L^{\infty }(0,T;L^{2}(\Omega_{i}^{\varepsilon }))}^{2}+\sum_{i=1}^{2}\|\nabla (u_{i}^{\varepsilon }-u_{i}^{1}){\|}_{L^{2}{\left(0,T;L^{2}(\Omega_{i}^{\varepsilon })\right)}^{2}}^{2}⩽{\text{Err}}_{12}(\varepsilon ),\\ & {\text{Err}}_{12}:=\frac{2}{\min\left(\frac{1}{2},\frac{1}{2K_{\text{emb}}},\gamma \right)}\left(\alpha_{10}{\text{Err}}_{11}(\varepsilon )+{\text{Err}}_{10}(\varepsilon )\right)=O(\varepsilon ), \end{array}$$

which finishes the proof.

## 7 DISCUSSION

Compared with previous results in the literature on multiscale diffusion equations, in the paper, we derived the macroscopic bidomain model that is advantageous for numerical simulation; we first proved the homogenization result supported by residual error estimate of the asymptotic corrector due to the nonlinear transmission condition at the microscopic level, which appears to describe interface chemical reactions.

For further generalization of the obtained result, we suggest to consider the case of connected‐disconnected domains $\Omega_{1}^{\varepsilon }$ and $\Omega_{2}^{\varepsilon }$. While in the connected domain $\Omega_{1}^{\varepsilon }$ the uniform extension is applicable, the disconnected domain $\Omega_{2}^{\varepsilon }$ allows a discontinuous Poincaré estimate (see Kovtunenko and Zubkova^21^).

## CONFLICT OF INTEREST

This work does not have any conflicts of interest.
